# Risk factors and injury prevention strategies for overuse injuries in adult climbers: a systematic review

**DOI:** 10.3389/fspor.2023.1269870

**Published:** 2023-12-12

**Authors:** Andrew Quarmby, Martin Zhang, Moritz Geisler, Tomas Javorsky, Hendrik Mugele, Michael Cassel, Justin Lawley

**Affiliations:** ^1^ Sports Medicine & Sports Orthopaedics, University of Potsdam, University Outpatient Clinic, Potsdam, Germany; ^2^ Department of Sports Science, Division of Performance Physiology and Prevention, University of Innsbruck, Innsbruck, Austria; ^3^ EURAC Research, Institute for Mountain Emergency Medicine, Bolzano, Italy

**Keywords:** climbing, bouldering, overuse injuries, risk factors, injury prevention, systematic reveiw, climbing injuries

## Abstract

**Introduction:**

Climbing is an increasingly popular activity and imposes specific physiological demands on the human body, which results in unique injury presentations. Of particular concern are overuse injuries (non-traumatic injuries). These injuries tend to present in the upper body and might be preventable with adequate knowledge of risk factors which could inform about injury prevention strategies. Research in this area has recently emerged but has yet to be synthesized comprehensively. Therefore, the aim of this study was to conduct a systematic review of the potential risk factors and injury prevention strategies for overuse injuries in adult climbers.

**Methods:**

This systematic review was conducted in accordance with the PRISMA guidelines. Databases were searched systematically, and articles were deemed eligible based upon specific criteria. Research included was original and peer-reviewed, involving climbers, and published in English, German or Czech. Outcomes included overuse injury, and at least one or more variable indicating potential risk factors or injury prevention strategies. The methodological quality of the included studies was assessed with the Downs and Black Quality Index. Data were extracted from included studies and reported descriptively for population, climbing sport type, study design, injury definition and incidence/prevalence, risk factors, and injury prevention strategies.

**Results:**

Out of 1,183 records, a total of 34 studies were included in the final analysis. Higher climbing intensity, bouldering, reduced grip/finger strength, use of a “crimp” grip, and previous injury were associated with an increased risk of overuse injury. Additionally, a strength training intervention prevented shoulder and elbow injuries. BMI/body weight, warm up/cool downs, stretching, taping and hydration were not associated with risk of overuse injury. The evidence for the risk factors of training volume, age/years of climbing experience, and sex was conflicting.

**Discussion:**

This review presents several risk factors which appear to increase the risk of overuse injury in climbers. Strength and conditioning, load management, and climbing technique could be targeted in injury prevention programs, to enhance the health and wellbeing of climbing athletes. Further research is required to investigate the conflicting findings reported across included studies, and to investigate the effectiveness of injury prevention programs.

**Systematic Review Registration:**

https://www.crd.york.ac.uk/, PROSPERO (CRD42023404031).

## 1. Introduction

Participation in climbing is growing rapidly, especially given its recently attained status as an Olympic sport (1). There are several unique disciplines of climbing, including traditional and sport climbing (practised outdoor), bouldering and lead climbing (practised indoor), and ice climbing, which is also practised outdoor, but indoor ice walls are available (2, 3). Each discipline is known to have its own specific performance demands and risk of injury (4, 5). Injury incidence rates for both traumatic and overuse injuries have been reported around 4.2 injuries per 1,000 climbing hours (6), indicating a similar injury risk profile to sports such as baseball and handball (7). The point prevalence of all injuries in climbers has been reported at 22.8% (8), whilst the one year prevalence of rock-climbing injuries appears to be around 50% (9) The majority of overuse injuries seem to occur in the upper extremities, whereas lower-extremity injuries are more commonly associated with falls (9). Acute lower-extremity injury seems to be particularly prevalent in bouldering, whereby nearly two-thirds of injuries treated in an emergency department and obtained whilst bouldering were located in the lower extremities (10). Injuries in climbing can be classified as both acute and overuse. Acute injuries are typically related to falling or environmental exposure such as rock falls, whereas persistent overuse injuries arise due to repetitive stress without adequate recovery, where one clear and exact traumatic cause for pain or structural deficit cannot be identified (11). Some injuries occur whilst overstraining in a single move, for example a finger pulley rupture when exerting high levels of force in a crimp grip against a hold (11). Such injuries would typically be defined as acute in nature, although the effects of preceding repetitive overuse and fatigue on the injured tissue cannot be ruled out. Most injuries in climbing are thought to be overuse in origin, with up to 93% of injuries defined as such (6). It would therefore appear pertinent to categorize injury risk as either traumatic or overuse, considering that the aetiology and risk factors associated with each category are known to be distinct (5, 6, 12). Injury prevention strategies and risk factor mitigation for traumatic injuries has mainly focused on adequate safety standards and training, equipment use, and type of climbing (12), whereas risk factors for overuse injuries seem more related to appropriate load management and training programming, particularly relating to the upper extremities (5, 13). A previous systematic review by Woollings et al., (2015) found that age, increasing years of climbing experience, higher climbing grade, high chronic training loads, and participating in lead climbing are potential risk factors for injury in sport climbing and bouldering (5). However, this analysis included both traumatic and overuse injuries. Concentrating solely on overuse injuries may be more insightful for practitioners, as some of the risk factors are likely modifiable and related to physical training programming (5, 14). Since the review by Woollings et al., (2015) literature in this area has been reviewed critically (13, 14) but not systematically, and research interest has grown significantly in recent years. An updated systematic review of the literature is therefore appropriate, to revise and synthesize existing knowledge. Moreover, this systematic analysis should identify more specific risk factors and thus support the development of injury prevention strategies to reduce overuse injuries in climbers. This knowledge is vital for coaches, clinicians, and the athletes themselves as more and more individuals are likely to push the limits of training in the pursuit of Olympic gold. Therefore, the aim of this study was to conduct a systematic review of the literature, relating to risk factors and injury prevention strategies for overuse injuries in adult climbers.

## 2. Methods

This systematic review was pre-registered in the international prospective register of systematic reviews (PROSPERO) (ID: CRD42023404031). Additionally, the review was conducted in accordance with the preferred reporting items for systematic reviews and meta-analyses (PRISMA) guidelines (15) (see Supplementary Material File S1).

### 2.1. Sources

The databases (PubMed, Web of Science and the Cochrane Library) along with the websites (The International Federation of Sport Climbing (https://cdn.ifsc-climbing.org/index.php/home-mobile), The International Rock Climbing Research Association (IRCRA) (https://www.ircra.rocks), UIAA—The International Climbing and Mountaineering Federation (https://theuiaa.org), The Beta Angel Project (https://beta-angel.com), and The Crag (https://www.thecrag.com/home) were searched for studies addressing risk factors and injury prevention of overuse injuries in climbers. The search date was 1st March 2023. The bibliographies of included studies were also searched for further relevant publications.

### 2.2. Search strategy

The key terms of “climbing”, “injury”, “risk factors”, and “injury prevention” were combined with the Boolean Operators “AND”/”OR” to search the selected databases. Truncation of search terms and MeSH terms were applied, to maximize the reach of the search. An example of the search strategy conducted in the database PubMed can be seen in Table 1, and the search strings for additional databases can be found in Supplementary Material File S2. Identified studies were exported into an electronic reference manager (Mendeley Desktop 1.19.8), and duplicates were removed semi-automatically with manual checking. The eligibility of identified records was then determined according to strict eligibility criteria.

**Table 1:** Details of search strategy conducted in pubMed.

Category	Terms
Climbing	[“Climb*"(Title/Abstract)] OR [“Boulder*"(Title/Abstract)] OR [“Mountaineering"(MeSH Terms)]
Injury	[“Wounds and injuries"(MeSH Terms)] OR [“Athletic Injuries"(MeSH Terms)] OR [“Injur*"(Title/Abstract)] OR [“Overuse"(Title/Abstract)]
Risk factors	[“Risk Factors"(MeSH Terms)] OR [“Protective Factors"(MeSH Terms)]
Injury prevention	[“Prevention Program” (Title/Abstract)] OR [“Train*” (Title/Abstract)]
(Human subjects)	NOT (Animals)

Categories of “climbing”, “injury” and (“risk factors” OR “injury prevention”) were combined with the Boolean Operator “AND”.

### 2.3. Eligibility criteria

Studies were included for analysis based upon the following criteria: (1) Original data published in peer-reviewed journals, (2) Adult climbers (mean age of sample >18 years old) at all levels and in all disciplines of climbing, (3) Study designs should be prospective, cross-sectional, retrospective, cohort, randomized controlled trials, case-control or case-series, (4) Published in the language of English, German, or Czech, (5) Studies should investigate overuse injuries (studies exclusively investigating acute/traumatic injuries were excluded), and additionally at least one potential risk factor or injury prevention strategy. Solely epidemiological studies or investigations into conservative treatment, injections, surgery and rehabilitation of injuries were excluded.

### 2.4. Selection process

Potential studies were screened independently by two reviewers and included according to the aforementioned eligibility criteria. Initially, titles and abstracts of identified studies were screened for eligibility. Upon inclusion, the full-text articles of studies were sought for further screening, and disagreements regarding study inclusion were arbitrated by a third author.

### 2.5. Data collection

Relevant parameters were manually extracted from the included studies and entered into a single table. Data was extracted for study design, participant characteristics and sample size, injury definition and incidence/prevalence, types of overuse injuries identified, risk factors and/or injury prevention strategies studied, and the results of associations between risk factors/prevention strategies and injury with statistical findings. In some cases, studies investigated all forms of injury occurrence including overuse and traumatic aetiologies. Where possible, overuse injuries were isolated and identified in relation to associated risk factors and prevention strategies. Reported climbing grades of participants were converted into the IRCRA comparative grading scale (16), to allow for easier comparison and interpretation of the included samples. To assess the methodological quality of the included studies, two independent reviewers conducted the Downs and Black questionnaire (17). The checklist scores studies out of 32 points and is referred to as “Study Quality Score” (SQS; x/32) in the results and discussion.

### 2.6. Data synthesis

The included studies were highly heterogenous in terms of objectives, methodology, and outcomes, and thus, a meta-analysis would not have been appropriate. Therefore, the data was synthesized descriptively, whereby trends in risk factors and prevention measures were interpreted qualitatively with reference to the methodological quality of the identified studies. Risk factors and injury prevention strategies were grouped into “modifiable” and “nonmodifiable” to assist with interpretation of the findings.

## 3. Results

### 3.1. Identification of studies

**Figure 1:**
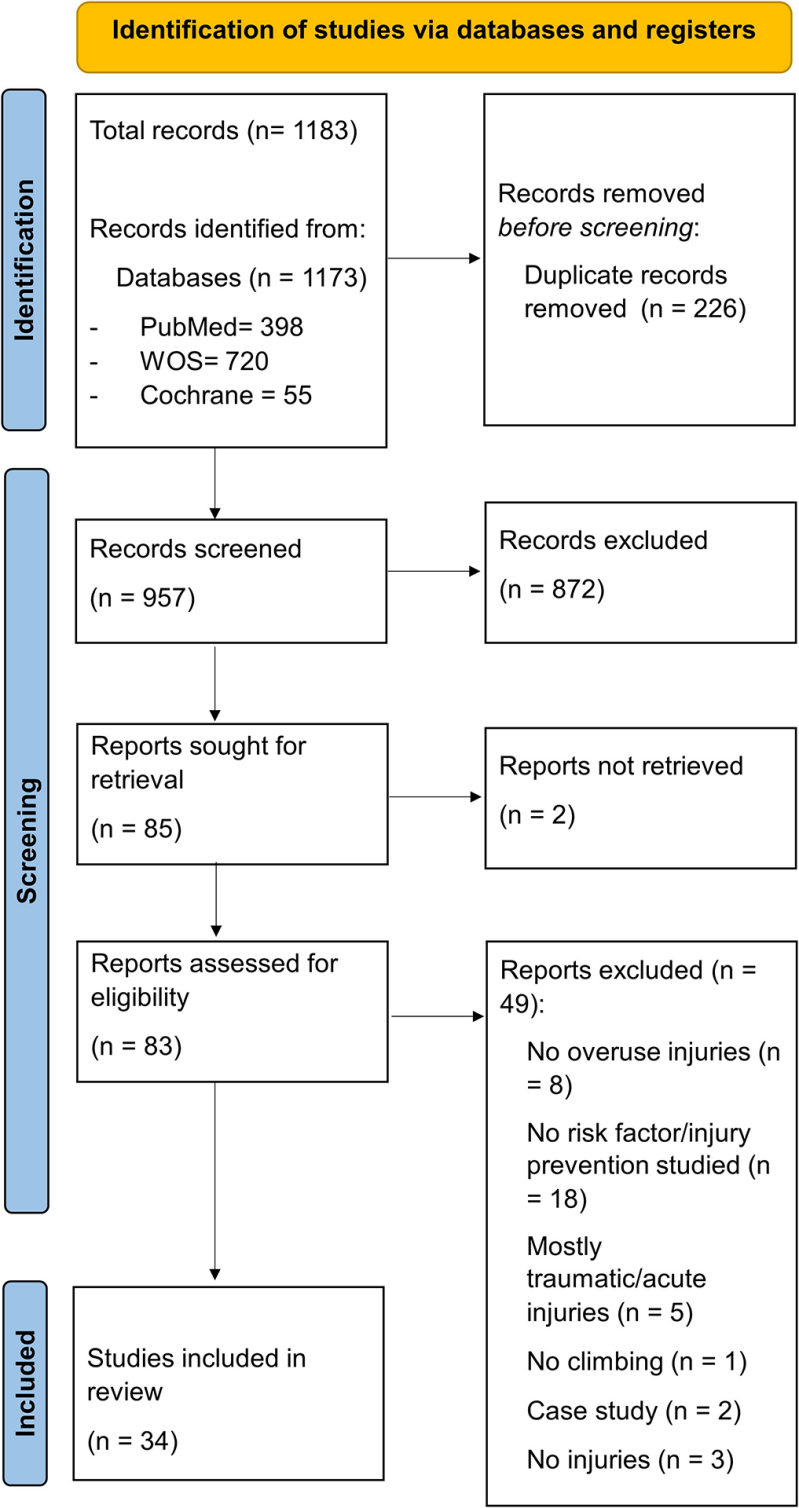
PRISMA flow diagram.

The results of the study selection process can be seen in Figure 1. Overall, 1,183 records were identified for screening. A total of 83 full-text reports were assessed for eligibility, of which 49 were excluded mainly because a risk factor wasn't studied, or no overuse injuries were mentioned. After complete screening, 34 studies were included in the final analysis.

### 3.2. Methodological quality assessment

The quality of included studies according to the Downs and Black criteria ranged from 8 to 20 out of a possible 32 points (mean: 14.4 points), indicating a large range of study quality (see Table 2). The quality of most studies overall was quite low, as 64.7% of studies were cross-sectional designs. The remainder of the studies were prospective designs plus two randomized controlled trials, and these studies generally obtained higher quality scores. The majority of studies performed particularly poorly in ratings of participant blinding (an inherent issue in many areas of sports medicine), randomization, and control of confounding factors which could have influenced outcomes.

**Table 2:** Methodological quality assessment of the included studies according to downs and black.

Study author name & year	Q1	Q2	Q3	Q4	Q5	Q6	Q7	Q8	Q9	Q10	Q11	Q12	Q13	Q14	Q15	Q16	Q17	Q18	Q19	Q20	Q21	Q22	Q23	Q24	Q25	Q26	Q27	Downs black score (/32)
Auer et al. (18)	1	1	1	0	0	1	1	0	0	1	1	1	0	0	0	1	1	1	0	1	0	1	0	0	0	1	5	19
Backe et al. (6)	1	1	1	0	1	1	1	0	1	1	0	0	0	0	0	0	0	1	0	1	0	0	0	0	0	1	5	16
Beeler et al. (19)	1	1	1	0	0	1	1	0	1	1	0	0	0	0	0	1	1	1	0	1	0	1	0	0	0	0	5	17
Bollen et al. (20)	0	0	0	0	0	0	0	0	1	0	0	0	0	0	0	1	0	1	0	0	0	0	0	0	0	1	5	9
Buda et al. (21)	1	1	1	0	0	1	1	0	0	1	0	0	0	0	0	0	0	1	0	1	1	1	0	0	0	0	5	15
Carmeli et al. (22)	1	1	1	0	0	1	1	0	0	1	0	0	0	0	0	0	0	1	0	1	0	0	0	0	0	0	5	13
Cobos-Moreno et al. (23)	1	0	1	0	0	1	1	0	1	1	0	0	0	0	0	1	0	1	0	0	1	1	0	0	0	1	5	16
Gerdes et al. (24)	1	1	1	0	0	1	1	0	0	1	0	0	0	0	0	0	0	1	0	1	1	1	0	0	0	0	5	15
Grønhaug et al. (25)	1	1	1	0	1	1	0	0	0	0	0	0	0	0	0	0	0	0	0	1	1	1	0	0	0	0	5	13
Grønhaug et al. (26)	1	1	1	0	1	1	1	0	0	1	0	0	0	0	0	0	0	1	0	0	1	1	0	0	0	0	5	15
Jones et al. (9)	1	1	1	0	1	1	1	1	0	1	0	0	0	0	0	0	0	1	0	1	1	0	0	0	1	0	1	13
Jones et al. (27)	1	1	1	0	0	1	1	0	1	1	1	1	0	0	0	1	1	1	0	1	1	1	0	0	0	0	5	20
Josephson et al. (28)	1	1	1	0	0	1	1	0	0	1	0	0	0	0	0	0	0	1	0	1	0	1	0	0	0	0	5	14
Killian et al. (29)	1	1	1	0	0	1	0	0	0	0	0	0	0	0	0	0	0	1	0	0	0	0	0	0	0	1	5	11
Kozin et al. (30)	1	1	1	1	1	1	1	0	0	1	0	0	0	0	0	0	0	1	0	1	1	1	1	0	0	0	5	18
Kozin et al. (31)	1	1	1	1	1	1	1	0	0	1	0	0	0	0	0	0	0	1	0	1	1	1	1	0	0	0	5	18
Lion et al. (32)	1	1	1	0	0	1	1	0	1	0	0	0	0	0	0	1	0	1	0	1	0	0	0	0	0	0	5	14
Logan et al. (33)	1	1	1	0	0	1	0	0	0	1	0	0	0	0	0	0	0	1	0	0	0	1	0	0	0	0	5	12
Lutter et al. (34)	1	1	1	0	0	1	1	0	1	1	0	0	0	0	0	1	1	1	0	1	0	1	0	0	0	0	5	17
Lutter et al. (35)	1	0	1	0	0	1	1	0	1	0	1	1	0	0	0	1	0	1	0	1	1	1	0	0	0	0	2	14
Nelson et al. (36)	1	1	1	0	0	1	1	0	1	1	0	1	0	0	0	1	0	1	0	1	0	0	0	0	0	0	5	16
Neuhof et al. (37)	1	1	1	0	1	1	1	0	0	0	0	0	0	0	0	0	0	1	0	1	1	1	0	0	0	0	5	15
Orth et al. (38)	0	1	1	0	0	1	1	0	1	0	0	0	0	0	0	1	0	1	0	1	1	0	0	0	1	0	5	15
Paige et al. (39)	1	0	0	0	0	1	0	0	0	0	0	0	0	0	0	0	0	0	0	0	0	1	0	0	0	0	5	8
Pieber et al. (40)	1	1	1	1	0	1	1	0	0	1	0	0	0	0	0	0	0	1	0	0	1	1	0	0	0	0	5	15
Rohrbough et al. (41)	1	1	1	0	0	1	1	0	1	1	0	0	0	0	0	0	0	1	0	0	0	0	0	0	0	0	2	10
Runer et al. (2)	1	1	1	0	1	1	1	0	0	1	0	0	0	0	0	0	0	1	0	0	1	1	0	0	0	0	5	15
Schäfer et al. (42)	0	0	0	0	0	1	0	0	1	0	0	0	0	0	0	1	0	1	0	0	1	0	0	0	0	1	5	11
Schöffl et al. (43)	1	1	1	0	0	1	1	0	1	1	0	0	0	0	0	1	0	1	0	1	1	1	0	0	0	1	5	18
Shahram et al. (44)	1	1	0	0	0	1	0	0	0	1	0	0	0	0	0	0	0	1	0	0	0	1	0	0	0	0	5	11
Stelzle et al. (45)	1	1	0	0	0	1	0	0	1	0	0	0	0	0	0	0	0	1	0	1	0	0	0	0	0	1	5	12
van Middelkoop et al. (8)	1	1	1	0	1	1	1	0	1	1	0	0	0	0	0	0	1	1	0	1	1	1	0	0	0	1	5	19
Wright et al. (46)	0	1	0	0	0	1	1	0	0	1	0	0	0	0	0	0	0	1	0	0	0	0	0	0	0	0	5	10
Zielinski et al. (47)	1	1	1	0	0	1	1	0	0	1	0	0	0	0	0	0	0	1	0	1	1	1	0	0	0	0	5	15

### 3.3. Participants

The characteristics of all included participants across all studies can be seen in Supplementary Material File S3 in the “sample characteristics” column. A total of 10,049 participants were included within this systematic review across 34 studies. The average age of all participants was 30.2 years (ranging from 19 to 54 years). Males were disproportionately represented when considering all studies. Regarding climbing discipline, participants could be categorized as follows: rock climbers (nine studies), sport climbers (eight studies), mixed discipline (13 studies), boulderers [two studies (18, 28)], and ice climbers [one study (2)]. IRCRA climbing grades scored an average of 17.4 in all studies (intermediate to advanced level) and ranged from 1 (lower grade) to 29 (higher elite).

### 3.4. Injury prevalence and incidence

The prevalence and incidence of injuries described within the individual studies can be found in Table 3. The reported numbers are extremely varied, probably due to the differences in populations and methodologies across the 34 studies. The incidence proportion ranged from 26 injuries per 100 participants to 300 injuries per 100 participants, whereas point/time prevalence ranged from 15% to 81%. These injury rates range across a diverse period from 6 months to whole career. Fifteen of the included studies investigated climbing injuries at specific anatomical sites, namely foot injuries/alterations (21, 23, 29), back pain (47), injuries of the shoulders (19, 30), elbow injuries (31), the fingers and hands (22, 32, 34, 43), Duputryen's disease (33), and upper extremity injuries (8, 36, 41). The remaining 19 studies investigated climbing injuries more broadly, and nine of these studies contained clear definitions of overuse injury within them (6, 9, 18, 25, 27, 35, 39, 40, 45, 46). Upper extremity injuries appear to be the most prevalent and well-studied across all included investigations.

**Table 3:** Injuries and epidemiological data from included studies.

Study author name & year	Injury prevalence, incidence rates (IR) or incidence proportions (IP)
Auer et al. (18)	15% incurred overuse injury over 12 months
Backe et al. (6)	IR of 4.2 injuries per 1,000 h of climbing
Beeler et al. (19)	71% career prevalence of shoulder pain
Bollen et al. (20)	Not reported
Buda et al. (21)	Not reported
Carmeli et al. (22)	Not reported
Cobos-Moreno et al. (23)	73.59% prevalence of foot injuries or alterations
Gerdes et al. (24)	Career IP of 131 per 100 participants
Grønhaug et al. (25)	IP of 58 injuries per 100 participants over past 6 months
Grønhaug et al. (26)	IP of 58 injuries per 100 participants over past 6 months
Jones et al. (9)	IP of 50.2 injuries per 100 participants over past 12 months
Jones et al. (27)	IP of 137 per 100 participants over 12 months
Josephson et al. (28)	IP 103 per 100 participants (outdoor climbing), IP 127 per 100 participants (indoor climbing)
Killian et al. (29)	81% point prevalence of pain/discomfort in feet
Kozin et al. (30)	Shoulder injuries: IR of 3.2 per 1,000 athlete exposures in control group vs. IR of 0.5 per 1,000 athlete exposures in injury prevention training group
Kozin et al. (31)	Elbow injuries: IR of 1.8 per 1,000 athlete exposures in control group vs. IR of 0.5 per 1,000 athlete exposures in injury prevention training group
Lion et al. (32)	IP of 67.4 hand injuries per 100 participants in last 3 years
Logan et al. (33)	19.5% point prevalence of Duputryen's disease
Lutter et al. (34)	IP of 93.5 per 100 participants
Lutter et al. (35)	IP of 94.4 overuse injuries per 100 participants over 3 year period
Nelson et al. (36)	IP of 90 per 100 participants for upper extremity injuries
Neuhof et al. (37)	IP of 28.5 per 100 participants
Orth et al. (38)	Not reported
Paige et al. (39)	IP of 63 injuries per 100 participants over last five years
Pieber et al. (40)	Career IP of 194 injuries per 100 participants
Rohrbough et al. (41)	Career IP of 300 injuries per 100 participants
Runer et al. (2)	IR of 9.8 injuries per 1,000 exposure hours over one winter season
Schäfer et al. (42)	Not reported
Schöffl et al. (43)	Finger stress reactions in 8/10 national level climbers and 3/10 recreational climbers over fiver-year period
Shahram et al. (44)	70% of climbers experienced injury
Stelzle et al. (45)	Career IP of 30 overuse injuries per 100 participants
van Middelkoop et al. (8)	IP of 13.04 per 1,000 climbing hours over one year
Wright et al. (46)	IP of 44 overuse injuries per 100 participants
Zielinski et al. (47)	IP of 26 mild back pain cases per 100 participants

### 3.5. Risk factors and injury prevention strategies

A total of 73 risk factors or injury prevention strategies were studied in the 34 included studies. For reasons of brevity, only the most prevalent of them are presented in the results section, divided into modifiable and nonmodifiable risk factors, though details of the remaining findings can be found in Supplementary Material File S3.

### 3.6. Modifiable risk factors and injury prevention strategies

#### 3.6.1. Body weight and body mass index (BMI)

Eight studies researched body weight and/or BMI (6, 8, 18, 26, 28, 32, 35, 37). Of these, three prospective studies (1–3 years in length) with relatively high study quality scores (SQS) (17/32–19/32) found no association between body weight or BMI and climbing injury (8, 18, 35). Additionally, three cross-sectional studies also showed no associated risk of climbing injury with BMI (26, 28, 37), whilst one cross-sectional study with a study quality score of 16/32 did indicate increased risk of injury with increased BMI (6). Furthermore, a single study determined that increased BMI was associated with a higher risk of hand injuries, although the data was cross-sectional (SQS: 14/32) (32).

#### 3.6.2. Type of climbing

A total of 11 studies investigated the type of climbing and associations with injury (6, 8, 9, 24, 25, 27, 28, 39, 44, 46, 47). Overall, three studies indicated that bouldering as a climbing activity was associated with an increased risk of injury, when compared with other forms of climbing (6, 46, 47). In addition, one further study found that bouldering increased the risk of injury in a univariate analysis (*p* = 0.046), but not in a secondary multivariate analysis (8). Bouldering frequency was also associated with overuse injury in two further studies (9, 27). In two studies, outdoor injuries were more prevalent than indoors, namely 61% vs. 27% in boulderers (28), and 74% of outdoor male climbers (25). Two studies showed that traditional climbing was not associated with injuries, whilst other forms of climbing such as lead and sport were (27, 39). Finally, one study showed no associations between type of climbing and injury risk, although the study had a relatively low SQS and sample size (11/32; *n* = 50) (44).

#### 3.6.3. Climbing volume

Eight studies measured climbing volume in some form, as a potential risk factor for injury (6, 8, 21, 22, 35–37, 48). A single 5-year prospective study with a relatively high SQS (18/32) suggested that hours of training per week and training units per week were significantly associated with finger stress reactions (48). This is contradicted by evidence in two different longitudinal prospective studies (SQS: 14/32 and 19/32), showing that there were no significant associations between climbing time per month/per week and the development of a climbing injury (8, 35). Five cross-sectional investigations revealed an increased risk of injury with increased climbing volume (6, 21, 22, 36, 37), in particular for recurrent ankle sprains potentially relating to chronic ankle instability (21), and injures of the wrist/fingers (22). Nelson et al., (2017) reported that the odds of sustaining an injury in people climbing every week were 2.49 times higher (95% CI: 1.27–4.90) compared to those who climb at most once a month (36).

#### 3.6.4. Climbing intensity

A total of 21 studies investigated climbing intensity and its relationship to injury, usually measured by climbing grade or level of climbing (e.g., intermediate vs. elite climbers) (2, 8, 9, 19, 21, 23, 25, 27, 29, 32, 33, 35–37, 40, 42, 44–48). Except for one prospective study (SQS: 14/32) (35), and a retrospective survey studying specifically injuries of the foot (SQS: 11/32) (29), the remaining 19 studies indicated some level of association between climbing intensity and an increased risk of injury, and the direction of this relationship was linear and positive (i.e., increased intensity = increased injuries). This evidence is supported by two longitudinal prospective studies (SQS: 18/32 and 19/32), specifically for finger stress injuries (48), and injuries of the entire upper extremity (8). Higher climbing intensity was associated with injuries in specific anatomical areas of the foot in three cross-sectional studies (21, 23, 33), the low back (47), and with degenerative changes in the shoulder (19). A single prospective study over one winter season highlighted that intermediate ice climbers were more likely to get injured than advanced ice climbers (2), however this finding is likely specific to ice climbers and probably alludes to the prevalence of traumatic injuries in the intermediate cohort as a result of lower skill.

#### 3.6.5. Strength and conditioning

Nine studies researched strength and conditioning measures as ether a risk factor or preventative strategy against climbing injuries (8, 22, 28, 30–32, 38, 42). Two studies reporting from the same randomized controlled trial (SQS: 18/32) showed that an injury prevention program based upon closed chain eccentric and strength exercises performed 3–4 times per week for one year, could reduce the likelihood of shoulder injuries (30) and elbow injuries (31). Additionally, prospective evidence (SQS: 14) suggests that weight training can reduce injuries in boulderers, although regular yoga practice had no positive effect (28). Cross-sectional evidence showed that the injured hand in climbers had weaker grip strength than the contralateral hand (22). An additional cross-sectional investigation adds weight to these findings (38) (SQS: 15/32), illustrating 7% mean deficits in maximal voluntary isometric contraction of the previously injured finger flexors. In contrast, prospective data (SQS: 19/32) indicates that higher strength of the middle finger and campus board training of the fingers is predictive of injury in the upper extremities (8). A different one-year prospective study (SQS: 19/32) found that fingerboard training was not associated with an increased risk of injury in boulderers (18). A single study examined cardiovascular training as preventive measure against hand injuries, and found no significant association (32). Finally, a single study discussed weak spinal musculature, strength training, and pull-up technique as indicators for climbing injury (42).

#### 3.6.6. Other prevention measures

Two longitudinal prospective studies (SQS: 19/32) found that finger taping is not an effective intervention for the prevention of injuries in boulderers (18, 28). Meanwhile, one of these prospective studies indicated that wrist taping might have a protective effect (28). Three studies (two of which were prospective designs) indicated that performing a warm-up has no impact on the prevalence of climbing injuries (8, 28, 32), whilst a separate prospective study (SQS: 19/32) actually reported increased odds of injury when conducting a finger-specific warm-up (18) though this finding was attributed to confounding variables not measured within the study design. Performing a cool-down appeared to have no effect on injury in a single cross-sectional study (32), whilst a prospective study (8) (SQS: 19/32) showed that cooling-down was associated with increased risk of injury. Stretching was non-protective against climbing injury in two studies (28, 32). Two studies discussed the repetitive use of a “crimp grip” as a potential risk factor for injuries of the hand and fingers (20, 45), and found a significant association with injury risk. A single study reported a strong correlation between shoe size reduction and neurological symptoms in the foot e.g., tingling, although shoe size reduction was not correlated with pain or discomfort (SQS: 11/32) (29). Lastly, hydration was investigated in a single-study and found not to be associated with injury risk in climbers (32).

### 3.7. Nonmodifiable risk factors

#### 3.7.1. Age and years of climbing experience

Age and years of experience are considered together, as they are likely somewhat colinear and may confound each other e.g., older people are more likely to have more years climbing experience. A total of 12 studies considered age as a risk factor for climbing injury, whilst ten studies examined years of climbing experience. Five studies identified older age as a significant risk factor for the development of climbing injury (6, 8, 22, 40, 41), whereby one of these studies was a higher-quality prospective study investigating upper extremity injuries only (8) (SQS: 19/32). Conflictingly, six studies (9, 18, 35–37, 46) including two prospective studies in boulderers (18) (SQS: 19/32) and a broad population of climbers (35) (14/32) found no significant relationship with increasing age and risk of climbing injury. Although, Lutter et al., (2019) only included a sample of four people in the 65 + years group (35). Interestingly, a single study indicated a higher risk of forearm bone marrow edema in younger climbing populations compared to their older peers, as measured via magnetic resonance imaging (34). Regarding years of climbing experience, two prospective studies propose that development of bone marrow edema in the hand is associated with increasing years of experience (34) (SQS: 17/32), and that male climbers who report more years of experience also have an increased risk of injury (35) (SQS: 14/32). Additionally, there is further cross-sectional evidence indicating increased risk of medial epicondylitis (41), injuries of the foot (21, 23), and general musculoskeletal injury (37), with increasing years of climbing experience. However, an additional four studies revealed no association between increasing years of climbing experience and injury risk (6, 9, 18, 28), whereby two of these were prospective investigations in boulder specific populations (18, 28) (SQS: 14/32; 19/32). It should be noted that the average age of participants in these two studies was relatively young (24.7–30 years).

#### 3.7.2. Sex

13 studies examined biological sex as a risk factor for climbing injury and provided conflicting results (6, 8, 9, 21, 22, 25, 28, 34, 35, 37, 40, 41, 46). A total of seven studies discovered a significant relationship between male sex and the risk of injury (6, 21, 22, 25, 35, 40, 46). One of these studies was conducted in a prospective design over a three-year period (35) (SQS: 14/32), and showed a male to female ratio of 3:1 in terms of injury rate, whereby males also had significantly higher climbing levels and years of experience which could be considered as confounding factors. One study added nuance to the results, indicating that male sex is indeed a risk factor for chronic elbow and finger injuries, but that females are at greater risk of chronic ankle injuries (SQS: 13/32) (25). In contrast, six studies suggested no relationship between biological sex and injury risk (8, 9, 28, 34, 37, 41), and three of these studies were prospective designs with relatively high quality scores (8, 28, 34) (SQS: 14/32–19/32). These studies were conducted to determine risk factors on hand bone marrow edema (34) and general injury risk in boulderers (28) and a broad climbing population (8).

#### 3.7.3. Previous injury

A total of four studies explored previous injury as a risk factor for the development of future injury or reinjury (18, 22, 27, 28). Three prospective studies with relatively high quality scores (SQS: 14/32–20/32) indicated a history of prior injury to be a significant predictor of future injury and/or reinjury (18, 27, 28). Jones et al., (2015) reported a 63% average probability for reinjury in climbers reporting a previous overuse injury, which was particularly evident for the fingers. In support of this data, Josephson et al., (2007) specifically indicated that a history of finger injury was predictive of a reinjury. A single cross-sectional study on 37 participants (22) (SQS: 13/32) contradicts the above findings, showing no relationship between past and current injury.

## 4. Discussion

The aim of this study was to conduct a systematic review of the potential risk factors and injury prevention strategies for overuse injuries in adult climbers. A total of 34 studies reporting on 73 risk factors or injury prevention strategies were included in the final analysis. The methodological quality of the included studies was variable (SQS: 8/32–20/32) and bias is likely to have impacted the findings in several studies. The methods of defining injury and risk factors or injury prevention strategies was extremely diverse, so overall conclusions drawn from the reviewed evidence should be treated with caution. For modifiable risk factors associated with injury, some key findings from the evidence can be stated. Strong evidence from prospective and cross-sectional studies indicates that increased climbing intensity is associated with an increased risk of injury, whereas the relationship between climbing volume and injury is much less clear. Strong evidence both prospectively and cross-sectionally suggest that BMI and/or body weight are not associated with an increased risk of injury. Regarding type of climbing, there is moderate evidence that bouldering might result in more injuries, when compared to other disciplines of climbing. An injury prevention strength training program was able to prevent elbow and shoulder injuries in two randomized controlled trials (RCT) (though on the same cohort), and there is weak evidence from cross-sectional studies that reduced grip/finger strength is associated with risk of injury. Limited evidence suggests that warm up/cool downs, stretching, taping and hydration have no relationship with climbing injury, whereas repetitive use of a “crimp grip” and shoe size reduction might be associated with injury. Considering nonmodifiable risk factors, the evidence for age/years of climbing experience and its association with injury was conflicting across studies. Evidence for the association between biological sex and climbing injury was equally conflicting. Meanwhile, strong prospective evidence suggests that previous injury is highly predictive of sustaining future climbing injuries.

### 4.1. Modifiable risk factors

#### 4.1.1. BMI/body weight not associated with overuse injury risk

In a previous systematic review by Woollings et al., (2015), the authors concluded that a higher BMI was likely to be associated with increased risk of injury in climbers. However, this conclusion was primarily based on data from Backe et al., (2009) due to its methodological rigor compared to other studies in the analysis. Since this review was published, three prospective investigations in a total of 1,138 climbers (8, 18, 35) have subsequently reported no association between increased BMI/body weight and risk of climbing injury. This strong evidence, supported by additional cross-sectional findings (26, 28, 37), suggests that practitioners working with climbing athletes should avoid strong recommendations on weight loss strategies in the pursuit of injury prevention. Furthermore, weight loss programs are commonly initiated in the pursuit of performance goals, however, associations between reduced BMI and improved climbing performance also seem limited (26). Therefore, such programs should be implemented with extreme caution, especially considering the risk of poor bone health and associated disorders in athletic populations (49). Hence, the health of the climbing athlete should be prioritized above all else (50). It should be acknowledged that weight loss strategies in the pursuit of performance goals will likely continue to be a staple in sports performance, especially in sports such as climbing where the strength to body weight ratio could still be assumed to influence performance for some athletes. Additionally, the data from Gronhaug (2019) showing no effect of low BMI on climbing performance is only cross sectional and retrospective, which weakens the findings substantially.

#### 4.1.2. Bouldering is potentially a greater risk factor for injury

Bouldering appears to be a risk factor for the development of climbing injury when compared to other disciplines such as lead climbing, as indicated by evidence in six studies (6, 8, 9, 27, 46, 47). However, the majority of the studies are cross-sectional in design, therefore strong conclusions cannot be drawn. Nonetheless, this contrasts with previous findings from Woollings et al., (2015) who suggested that lead climbing was associated with an increased risk of injury in climbers. The conflicting results can potentially be explained on two fronts. Firstly, some of the evidence in the current study has emerged since the publication of this previous systematic review. Secondly, the focus of the current study was overuse injuries, whereas the review from Woollings et al., (2015) also included all traumatic injuries by methodology. It could be speculated that lead climbing might result in more traumatic injuries due to the higher risk of larger falls, compared to bouldering which might be associated with a higher amount of overuse related injuries, and traumatic injuries to the leg/ankle during falling. Bouldering is typified by repetitive intense bouts of dynamic climbing, and this specific pattern of highly demanding effort may put climbers at a greater risk of developing an overuse injury (18). Practically, boulderers should be encouraged to take sufficient rest periods between intense bouts of climbing to allow for recovery and mitigate fatigue.

#### 4.1.3. A relationship between climbing volume and injury risk is unclear

The amount of time spent climbing (climbing volume) was shown to be associated with finger stress injuries in one prospective study (48), though the sample size was small with only 20 participants (SQS: 18/32). This is contrasted in two prospective studies (8, 35) (*n* = 198; *n* = 434), which showed no relationship between total climbing volume and general injury risk in climbers (SQS: 14/32; 19/32). It might be important to note that the average age of the sample in the study by Schöffl et al., (2007) (48) was much younger (20 to 21 years old) than in the other two prospective studies (32 to >65 years old), perhaps indicating that large climbing volume may be a risk factor for younger climbing athletes specifically. Despite further evidence from five cross-sectional studies suggesting an association between increased volume and injury risk (6, 21, 22, 36, 37), it is difficult to state that a clear relationship exists in the context of findings from this review. The conflicting results on training volume in the current study support previous conclusions published in a systematic review by Woollings et al., (2015). The discrepancy in findings from the included studies could be explained by variation in the methodologies of reporting training volume, whereby information collected in the form of questionnaires is known to be subject to recall and/or response bias. Future studies could incorporate wearable sensor technology to monitor training volume, which might enable more accurate measurement of time spent training (51). Additionally, it could be considered that training volume may also offer a protective stimulus against injury (52), which might also explain the paradoxical findings. When applied consistently, large training volumes will induce physiological adaptations in athletes which prepare them for their sport and competition, and therefore might actually assist in the prevention of injuries (52). Whilst it must be acknowledged that increased training volume has been suggested to be associated with injury risk in athletes (53), it has equally been debated whether sudden and rapid spikes in training volume may be responsible for the increased injury risk, as opposed to training volume when considered as a consistent variable over a period of time (54). The results of the current study and others (5, 9, 55), indicate that future studies should be conducted to investigate the relationship between training volume and climbing overuse injuries, with an enhanced focus on the quality of the data collected.

#### 4.1.4. Climbing intensity is associated with risk of overuse injury

Climbing intensity was usually measured indirectly via climbing level or grade, whereby a higher grade indicates a higher intensity. A total of 19 studies showed an association between increased climbing intensity and an increased risk of overuse injury (2, 8, 9, 19, 21, 23, 25, 27, 32, 33, 36, 37, 40, 42, 44–48), whereby two of the studies were prospective (8, 48) in a total of 454 climbers (SQS: 18/32 and 19/32). A single prospective study conflicted with this evidence (35) (SQS: 14/32), though this research included a much older population of athletes compared to other studies. This may have confounded the findings as older athletes generally reported lower climbing grades. The findings of the current study support previous work by Woollings et al., (2015), who also described a relationship between increased climbing intensity/grade and an increase in injury risk, and this association has been discussed in other reviews in the literature (14). High intensity training maintained over long periods of time (52) or when introduced abruptly during the training process (54) appears to increase the likelihood of sustaining an overuse injury in athletes, and this risk likely exists in climbers. This result underlines the need for training to be programmed and monitored in a sensible and accurate way, in accordance with currently known best practice in training periodization and planning (52, 56, 57). Approaches should emphasize a balanced training paradigm, which includes periods of intense training sessions to elicit the desired physiological adaptations, counteracted with “easier” sessions which allow for adequate recovery (52, 56). Research in this area specifically for climbers is notably scarce, and more studies are required with a focus on more valid methodological approaches for measuring climbing “intensity”. People working in climbing could adopt the “Climbing Intensity Score” (=climbing grade/level × climbing volume), as suggested by Logan et al., (2005). Such a score may provide a more comprehensive measurement of the total load experienced by climbing athletes and help to inform future climbing studies and load management strategies.

#### 4.1.5. Strength and conditioning for injury prevention

Two RCTs conducted on the same study cohort, reported that closed chain eccentric and strength exercises performed 3–4 times per week for one year, could reduce the likelihood of shoulder injuries (30) and elbow injuries (31) (SQS: 18/32). This is the first and seemingly only study which has implemented an injury prevention program in climbers and showed positive effects of a strength and conditioning program on injury reduction. Strength and neuromuscular training programs are broadly supported in the literature as an injury prevention modality in multiple sports (55), and it has been shown in a recent systematic review and meta-analysis that climbing-specific resistance training can also improve climbing performance (58). Therefore, it would seem appropriate to implement strength training programs in climbers. However, the results of the two included studies in this review had a relatively small sample size (*n* = 84) focusing on injuries of the elbows and shoulders (30, 31). Correspondingly, these findings cannot be applied to injuries of the fingers, which is known to be the most common site of injury in climbers (14). Furthermore, there is cross-sectional evidence in two studies that the injured hand is weaker than the contralateral healthy hand (22, 38), and an additional two prospective studies showed conflicting findings regarding finger strength and injury risk (8, 18). The prospective study by van Middelkoop et al., (2015) reported that increased strength of the middle finger and campus board training was actually predictive of injury risk. However, this might be understood as confounded noise in the data, whereby people with previous injuries to the fingers have adopted specific finger flexor training modalities in an attempt to prevent reinjury. This highlights the need for future RCTs investigating injury prevention programs specifically for the hand and fingers in climbers.

#### 4.1.6. Taping, warm up/cool down, and stretching mostly ineffective for injury prevention

Two high-quality prospective trials including a total of 658 climbers showed that taping did not protect against overuse injury risk (18, 28). Josephsen et al., (2007) did reveal that wrist taping could have a beneficial effect for boulderers, although confidence limits for the incidence rates indicated a weak effect. Taping is widely adopted in climbing gyms as means to prevent injury, but the results of this review cannot support its use. Warm-ups and cool-downs are commonly implemented in climbers to reduce the risk of overuse injury. However, based upon data from four studies (8, 18, 28, 32), two of which were prospective studies (8, 18), both warming up or cooling down had no protective effect against overuse injuries. The ritual of a warm-up or cool-down is likely to have other effects on athletic physiology, and there is some evidence that it may improve athletic performance (59). However, based upon the results of this review and others similar (5), traditional warm-ups or cool-downs cannot be recommended with the explicit goal of reducing overuse injury risk. Another commonly practiced injury prevention technique is stretching, usually performed prior to climbing in a static or dynamic manner. Two studies included in this review (28, 32) including one prospective investigation (28) indicate that stretching offers no protective effect against risk of overuse injury. This finding is commonly reported across other sporting disciplines (55) and supports results of a previous systematic review in climbing (5). Given evidence that static stretching doesn't confer a beneficial effect on muscle performance, and may even have a negative effect at longer duration stretch routines (>60 s) (60), it would seem problematic to advise climbers to engage with a stretching routine prior to training sessions and competition.

### 4.2. Nonmodifiable risk factors

#### 4.2.1. Relationship between age, years of climbing experience, and injury risk inconclusive

Results from this review indicate conflicting findings, when considering age and years of climbing experience as a risk factor for overuse injury. There is strong prospective evidence in a sample of 434 climbers that increasing age might exacerbate injury risk (8) (SQS: 19/32), which is contrasted by two prospective studies including a total of 704 climbers indicating no increased risk of injury with older age (18, 35) (SQS: 19/32, 14/32) (*n* = 229). Furthermore, two prospective studies showed a positive relationship between increased years of climbing experience and injury (34, 35), which conflicts with two prospective studies showing no relationship (18, 28) (*n* = 658). The mixed findings might be attributed to the large variety of injuries included within this review (see Table 3), whereby age and years of climbing experience might be a risk factor for certain injuries, but not for others. In a systematic review, Woollings et al., (2015) concluded that older age was a risk factor for injury in climbing, however, they also suggested that certain injuries may be more prevalent in younger populations when compared to older climbers. To this point, a recent prospective study included in this review indicated that younger climbers may be at a higher risk of bone marrow edema (34), although there were only 31 participants included in the study. This may be especially relevant for the physis of the proximal inter-phalangeal joint during adolescent growth, and practitioners should be vigilant for these issues in younger climbers. An additional consideration might be that older climbers tend to “self-moderate” the intensity of their climbing, by selecting lower grades as they age. This seems to be the pattern in the prospective analysis of Lutter et al., (2019), though this is only speculation and would require further study to verify this claim.

#### 4.2.2. Association between sex and injury risk conflicting

The findings relating biological sex to injury risk are also very conflicting. One prospective study with 198 participants showed that males were more likely to obtain an injury at a 3:1 ratio compared to females (35) (SQS: 14/32). However, in this sample males also reported higher climbing levels and years of experience when compared to females, which have also been discussed as risk factors for overuse injury. A cross-sectional study indicated that males are more at risk for chronic elbow and finger injuries, whereas females appear to have a higher risk of chronic ankle injuries (SQS: 13/32) (25). The author highlights that this may be due to climbing shoe design which is typically male-centric, therefore, shoes designed specifically for female feet should be developed and tested in future studies. In this same paper, male sex was seen to interact with bouldering grade, whereby a higher prevalence of chronic injuries was found in males with higher bouldering grades, however this interaction was not as obvious in females. In fact, the highest prevalence of injuries was amongst the male outdoor climbing group (74%), which is speculated to be reflective of the increased risk-taking behaviours of males when compared to females, especially when climbing outdoors. In contrast, three prospective studies with a total of 617 participants reported no differences for injury risk between the male and female sexes (8, 28, 34) (SQS: 14/32–19/32). Sex-specific differences in injuries have been revealed to some degree in team sports (61), though the differences seem marginal. It should also be noted that most of the studies included samples that were disproportionately male-biased, and therefore future research needs to be mindful of conducting research in female climbing populations. According to this review, there is conflicting evidence regarding sex-specific differences in risk factors for overuse injuries in climbers. In future studies, biological sex should be studied alongside other interacting factors, such as climbing grade and type of climbing e.g., indoors vs. outdoors, so as to reveal how these factors might coalesce and effect injury risk.

#### 4.2.3. Previous injury predicts future injury

Three relatively high quality prospective studies (18, 27, 28) (SQS: 14/32–20/32) identify previous injury as a risk factor for future injury or reinjury. This supports evidence in other sports (62), whereby the mechanism is supposed to occur in altered neuromuscular physiology associated with the injured anatomical site and potentially unresolved structural pathology in the local tissue. However, some studies included within the current review are not clear whether participants reinjured the same specific anatomical site that was injured previously, or whether injury risk is more generally heightened in individuals who have obtained previous injuries. These details should be the subject of future investigations. Nonetheless, it would be advised that climbers who have previously acquired an overuse injury should be aware of the increased risk of reinjury and follow the advice of their healthcare practitioner, and potentially implement a secondary injury prevention program with appropriate load management. Return to sport guidelines in climbing post-injury have yet to be deciphered, but there is some data available which could help inform decision-making (63, 64).

### 4.3. Limitations

It is important to acknowledge that this study has several limitations. Firstly, the primary limitation is that this systematic review can only make conclusions based upon the available data, and their chosen methodological approach. For example, all studies included in this review only examined the independent effects of chronological age, climbing experience, training volume and intensity, yet an overuse injury is likely the interactive effect of these factors. For example, the overall training load is a combination of volume and intensity, and an abrupt increase in training load is likely tolerable with a relatively low risk in well trained individuals, but a higher risk for overuse injury in untrained inexperienced climbers. Any conclusions on these interactive nuances cannot be made with the current level of evidence. Secondly, this systematic review focused on adult climbers and only studies researching this population were included. However, some studies also included participants younger than 18 years old, so this demographic was not entirely excluded from the analysis. Nevertheless, the mean age of all participants included was around 31 years old, so the large majority of climbers studied were very likely adults. Additionally, the aim of this review was to study overuse injuries only, as opposed to traumatic injuries. Several studies reported data on all types of climbing injuries, without specifically mentioning distribution of overuse or traumatic injuries within their research. Therefore, it is likely that some of the risk factors included in the analysis are related to traumatic injuries, which does limit the interpretation of the findings to some degree. Best efforts were made to isolate overuse injuries wherever possible. Future studies should attempt to categorize injuries more transparently so that better conclusions can be drawn. Finally, the participants included in this review were highly heterogenous, in terms of type of climbers, age, injuries, and the risk factors studied. This results in a large variation in the nature of our findings and makes it difficult to apply the information to specific groups. However, this methodology was chosen to obtain a broad range of data on climbing athletes, so as to synthesize the currently available data in a single review.

### 4.4. Conclusions

Within this systematic review, several risk factors and injury prevention strategies were identified for climbing athletes, some of which are modifiable. Modifiable risk factors are likely to be most relevant for coaches and clinicians working with climbers, as they can be changed and may prove as useful targets for injury prevention strategies. There is evidence that increased climbing intensity and bouldering are associated with a higher risk of overuse injury, and training for climbers should be planned and monitored in accordance with these findings. Climbing volume appears to be less relevant as a risk factor in general but might be a risk factor in specifically younger populations, and it would still seem pertinent to monitor training volume in addition to training intensity until future research can clarify this relationship. It is generally recommended that future prospective trials are required to validate the impact that training programming can truly have on injury risk reduction in climbers. Additionally, strength and conditioning training appears to be a successful strategy for mitigating injury risk in the shoulders and elbows. However, future intervention trials are necessitated to verify these results and to study prevention in other anatomical areas e.g., the fingers. BMI and body weight appear to have no relationship with overuse injury risk; therefore, aggressive weight loss programs are not useful in the pursuit of injury reduction. The nonmodifiable risk factors of age/years of experience and sex seem to have a conflicting relationship with overuse injury risk, therefore further research is required to clarify differences in climbing injury between younger and older climbing athletes, as well as males and females. A previous climbing injury appears to be a strong predictor of future injury. Climbing athletes who have suffered a prior overuse injury are recommended to complete a comprehensive rehabilitation program with a healthcare professional. This could potentially address associated deficits that might lead to future cases of injury. Finally, the risk factors and injury prevention strategies identified within the current literature are overwhelmingly related to physical therapeutics, training, load management, age, and sex. There is a paucity of information on psychosocial factors, sleep, and nutrition, although it is well documented that these aspects are likely to be associated with overuse injuries (65–67). Future climbing researchers are encouraged to investigate these variables in the context of overuse injury prevention in climbers, to shed more light on these issues and hopefully improve the health of climbing athletes.

## Data Availability

The original contributions presented in the study are included in the article/**Supplementary Material**, further inquiries can be directed to the corresponding author.

## References

[1] Lutter C El-Sheikh Y Schöffl I Schöffl V. Sport climbing: medical considerations for this new Olympic discipline. Br J Sports Med. (2017) 51(1):2–3. 10.1136/bjsports-2016-096871 27821387

[2] Runer A Lampl K Neunhäuserer D Runer F Frick N Seitlinger G A 1-year prospective analysis of ice climbing injuries. Clin J Sport Med Off J Can Acad Sport Med. (2017) 27(2):161–7. 10.1097/JSM.0000000000000326 27428673

[3] Woollings KY McKay CD Kang J Meeuwisse WH Emery CA. Incidence, mechanism and risk factors for injury in youth rock climbers. Br J Sports Med. (2015) 49(1):44–50. 10.1136/bjsports-2014-094067 25385168

[4] Saul D Steinmetz G Lehmann W Schilling AF. Determinants for success in climbing: a systematic review. J Exerc Sci Fit. (2019) 17(3):91–100. 10.1016/j.jesf.2019.04.002 31193395 PMC6527913

[5] Woollings KY McKay CD Emery CA. Risk factors for injury in sport climbing and bouldering: a systematic review of the literature. Br J Sports Med. (2015) 49(17):1094–9. 10.1136/bjsports-2014-094372 26009554

[6] Backe S Ericson L Janson S Timpka T. Rock climbing injury rates and associated risk factors in a general climbing population. Scand J Med Sci Sports. (2009) 19(6):850–6. 10.1111/j.1600-0838.2008.00851.x 19508652

[7] Gimigliano F Resmini G Moretti A Aulicino M Gargiulo F Gimigliano A Epidemiology of musculoskeletal injuries in adult athletes: a scoping review. Med. (2021) 57(10):1118. 10.3390/medicina57101118 PMC8539527 34684155

[8] van Middelkoop M Bruens ML Coert JH Selles RW Verhagen E Bierma-Zeinstra SMA Incidence and risk factors for upper extremity climbing injuries in indoor climbers. Int J Sports Med. (2015) 36(10):837–42. 10.1055/s-0035-1547224 25958937

[9] Jones G Asghar A Llewellyn DJ. The epidemiology of rock-climbing injuries. Br J Sports Med. (2008) 42(9):773–8. 10.1136/bjsm.2007.037978 18065444

[10] Muller M Heck J Pfluger P Greve F Biberthaler P Cronlein M. Characteristics of bouldering injuries based on 430 patients presented to an urban emergency department. Inj J Care Inj. (2022) 53(4):1394–400. 10.1016/j.injury.2022.02.003 35144805

[11] Sims LA. Upper extremity injuries in rock climbers: diagnosis and management. J Hand Surg Am. (2022) 47(7):662–72. 10.1016/j.jhsa.2022.01.009 35256226

[12] Rauch S Wallner B Strohle M Dal Cappello T Maeder MB Ströhle M Climbing accidents-prospective data analysis from the international alpine trauma registry and systematic review of the literature. Int J Environ Res Public Health. (2020) 17(1):203. 10.3390/ijerph17010203 PMC6981967 31892182

[13] Cole KP Uhl RL Rosenbaum AJ. Comprehensive review of rock climbing injuries. J Am Acad Orthop Surg. (2020) 28(12):e501–9. 10.5435/JAAOS-D-19-00575 32015250

[14] Jones G Johnson MI. A critical review of the incidence and risk factors for finger injuries in rock climbing. Curr Sports Med Rep. (2016) 15(6):400–9. 10.1249/JSR.0000000000000304 27841811

[15] Page MJ McKenzie JE Bossuyt PM Boutron I Hoffmann TC Mulrow CD The PRISMA 2020 statement: an updated guideline for reporting systematic reviews. BMJ. (2021) 372:n71. 10.1136/bmj.n71 33782057 PMC8005924

[16] Draper N Giles D Schöffl V Konstantin Fuss F Watts P Wolf P Comparative grading scales, statistical analyses, climber descriptors and ability grouping: international rock climbing research association position statement. Sports Technol. (2016) 8(3–4):88–94. 10.1080/1934618220151107081

[17] Downs SH Black N. The feasibility of creating a checklist for the assessment of the methodological quality both of randomised and non-randomised studies of health care interventions. J Epidemiol Community Heal. (1998) 52:377–84. 10.1136/jech.52.6.377 PMC1756728 9764259

[18] Auer J Schoffl VR Achenbach L Meffert RH Fehske K. Indoor bouldering-a prospective injury evaluation. WILDERNESS Environ Med. (2021) 32(2):160–7. 10.1016/j.wem.2021.02.002 33966976

[19] Beeler S Pastor T Fritz B Filli L Schweizer A Wieser K. Impact of 30 years’ high-level rock climbing on the shoulder: an magnetic resonance imaging study of 31 climbers. J Shoulder Elb Surg. (2021) 30(9):2022–31. 10.1016/j.jse.2020.12.017 33545338

[20] Bollen SR. Soft tissue injury in extreme rock climbers. Br J Sports Med. (1988) 22(4):145–7. 10.1136/bjsm.22.4.145 3228682 PMC1478743

[21] Buda R Di Caprio F Bedetti L Mosca M Giannini S. Foot overuse diseases in rock climbing: an epidemiologic study. J Am Podiatr Med Assoc. (2013) 103(2):113–20. 10.7547/1030113 23536501

[22] Carmeli E Shuruk S Sheklow SL Masharawi Y. Incidence of hand injuries in wall climbers: a comparison between adolescent adults and young adults. Biol Sport. (2002) 19(4):283–94.

[23] Cobos-Moreno P Astasio-Picado Á Gómez-Martín B. Epidemiological study of foot injuries in the practice of sport climbing. Int J Environ Res Public Health. (2022) 19(7):4302. 10.3390/ijerph19074302 35409982 PMC8998933

[24] Gerdes EM Hafner JW Aldag JC. Injury patterns and safety practices of rock climbers. J Trauma. (2006) 61(6):1517–25. 10.1097/01.ta.0000209402.40864.b2 17159699

[25] Grønhaug G. Self-reported chronic injuries in climbing: who gets injured when? BMJ Open Sport Exerc Med. (2018) 4(1):e000406. 10.1136/bmjsem-2018-000406 PMC6059297 30057779

[26] Gronhaug G. Lean and mean? Associations of level of performance, chronic injuries and BMI in sport climbing. BMJ Open Sport Exerc Med. (2019) 5(1):e000437. 10.1136/bmjsem-2018-000437 30687516 PMC6326274

[27] Jones G Llewellyn D Johnson MI. Previous injury as a risk factor for reinjury in rock climbing: a secondary analysis of data from a retrospective cross-sectional cohort survey of active rock climbers. BMJ Open Sport Exerc Med. (2015) 1(1):1–5. 10.1136/bmjsem-2015-000031 PMC5117049 27900114

[28] Josephsen G Shinneman S Tamayo-Sarver J Josephsen K Boulware D Hunt M Injuries in bouldering: a prospective study. Wilderness Environ Med. (2007) 18(4):271–80. 10.1580/06-WEME-OR-071R1.1 18076293

[29] Killian RB Nishimoto GS Page JC. Foot and ankle injuries related to rock climbing. The role of footwear. J Am Podiatr Med Assoc. (1998) 88(8):365–74. 10.7547/87507315-88-8-365 9735622

[30] Kozin S Cretu M Kozina Z Chernozub A Ryepko O Shepelenko T Application of closed kinematic chain exercises with eccentric and strength exercises for the shoulder injuries prevention in student rock climbers: a randomized controlled trial. Acta Bioeng Biomech. (2021) 23(2):159–68. 10.37190/ABB-01828-2021-01 34846050

[31] Kozin S Kozina Z Jagiello M Joksimovic M. Injury prevention of student rock climbers based on the formation of rational technique of movements: a randomized control trial. Phys Educ Students. (2021) 25(5):307–18. 10.15561/20755279.2021.0507

[32] Lion A van der Zwaard BC Remillieux S Perrin PP Buatois S. Risk factors of hand climbing-related injuries. Scand J Med Sci Sports. (2016) 26(7):739–44. 10.1111/sms.12505 26105683

[33] Logan AJ Mason G Dias J Makwana N. Can rock climbing lead to dupuytren’s disease? Br J Sports Med. (2005) 39(9):639–44. 10.1136/bjsm.2004.015792 16118302 PMC1725323

[34] Lutter C Hochholzer T Bayer T Schöffl V. Rock climbing-related bone marrow edema of the hand: a follow-up study. Clin J Sport Med Off J Can Acad Sport Med. (2018) 28(4):382–8. 10.1097/JSM.0000000000000463 28696958

[35] Lutter C Hotfiel T Tischer T Lenz R Schoffl V. Evaluation of rock climbing related injuries in older athletes. WILDERNESS Environ Med. (2019) 30(4):362–8. 10.1016/j.wem.2019.06.008 31668938

[36] Nelson CE Rayan GM Judd DI Ding K Stoner JA. Survey of hand and upper extremity injuries among rock climbers. Hand. (2017) 12(4):389–94. 10.1177/1558944716679600 28644933 PMC5484453

[37] Neuhof A Hennig FF Schöffl I Schöffl V. Injury risk evaluation in sport climbing. Int J Sports Med. (2011) 32(10):794–800. 10.1055/s-0031-1279723 21913158

[38] Orth D Slebioda N Cavada A van Bergen N Deschle N Hoozemans M. Persistent unilateral force production deficits following hand injury in experienced climbers: a reliability and retrospective injury study. Wilderness Environ Med. (2022) 34(1):22–30. 36517389 10.1016/j.wem.2022.10.001

[39] Paige TE Fiore DC Houston JD. Injury in traditional and sport rock climbing. Wilderness Environ Med. (1998) 9(1):2–7. 10.1580/1080-6032(1998)009[0002:IITASR]2.3.CO;2 11990177

[40] Pieber K Angelmaier L Csapo R Herceg M. Acute injuries and overuse syndromes in sport climbing and bouldering in Austria: a descriptive epidemiological study. Wien Klin Wochenschr. (2012) 124(11–12):357–62. 10.1007/s00508-012-0174-5 22661041

[41] Rohrbough JT Mudge KM Schilling RC. Overuse injuries in the elite rock climber. Med Sci Sports Exerc. (2000) 32(8):1369–72. 10.1097/00005768-200008000-00002 10949000

[42] Schäfer J Gaulrapp H Pförringer W. Acute and chronic overuse injuries in extreme sport-climbing. Sportverletz Sportschaden. (1998) 12(1):21–5. 10.1055/s-2007-993330 9592915

[43] Schoffl VR Schoffl I Schöffl VR Schöffl I. Finger pain in rock climbers: reaching the right differential diagnosis and therapy. J Sports Med Phys Fitness. (2007) 47(1):70–8. 17369801

[44] Shahram A Farzad A Reza R. A study on the prevalence of muscular-Skeleton injuries of rock climbers. Facta Univ Ser Phys Educ Sport. (2007) 5(1):1–7.

[45] Stelzle FD Gaulrapp H Pforringer W. Acute injuries and chronic overuse syndroms due to rock climbing on artificial climbing walls. Sportverletzung-sportschaden. (2000) 14(4):128–33. 10.1055/s-2000-8951 11199402

[46] Wright DM Royle TJ Marshall T. Indoor rock climbing: who gets injured? Br J Sports Med. (2001) 35(3):181–5. 10.1136/bjsm.35.3.181 11375878 PMC1724320

[47] Zieliński G Zięba E Wilkowicz W Byś A Ginszt M Libera O Influence of regular climbing on depression, generalized anxiety and lower back pain. Ann Agric Environ Med. (2021) 28(3):463–8. 10.26444/aaem/124189 34558271

[48] Schoffl VR Hochholzer T Imhoff AB Schoffl I. Radiographic adaptations to the stress of high-level rock climbing in junior athletes - a 5-year longitudinal study of the German junior national team and a group of recreational climbers. Am J Sports Med. (2007) 35(1):86–92. 10.1177/0363546506293256 16973900

[49] Hoenig T Ackerman KE Beck BR Bouxsein ML Burr DB Hollander K Bone stress injuries. Nat Rev Dis Prim. (2022) 8(1). 10.1038/s41572-022-00352-y 35484131 PMC13227457

[50] Turocy PS DePalma BF Horswill CA Laquale KM Martin TJ Perry AC National athletic trainers’ association position statement: safe weight loss and maintenance practices in sport and exercise. J Athl Train. (2011) 46(3):322–36. 10.4085/1062-6050-46.3.322 21669104 PMC3419563

[51] Sperlich B Aminian K Düking P Holmberg HC. Editorial: wearable sensor technology for monitoring training load and health in the athletic population. Front Physiol. (2020) 10:474046. 10.3389/fphys.2019.01520 PMC6960165 31969826

[52] Gabbett TJ. The training—injury prevention paradox: should athletes be training smarter and harder? Br J Sports Med. (2016) 50(5):273–80. 10.1136/bjsports-2015-095788 26758673 PMC4789704

[53] Sugimoto D Jackson SS Howell DR Meehan WP Stracciolini A. Association between training volume and lower extremity overuse injuries in young female athletes: implications for early sports specialization. Phys Sportsmed. (2019) 47(2):199–204. 10.1080/00913847.2018.1546107 30403911

[54] Windt J Zumbo BD Sporer B Macdonald K Gabbett TJ. Why do workload spikes cause injuries, and which athletes are at higher risk? Mediators and moderators in workload–injury investigations. Br J Sports Med. (2017) 51(13):993–4. 10.1136/bjsports-2016-097255 28274916

[55] Stephenson SD Kocan JW Vinod A V Kluczynski MA Bisson LJ. A comprehensive summary of systematic reviews on sports injury prevention strategies. Orthop J Sport Med. (2021) 9(10):23259671211035776. 10.1177/23259671211035776 PMC8558815 34734094

[56] Hamlin MJ Wilkes D Elliot CA Lizamore CA Kathiravel Y. Monitoring training loads and perceived stress in young elite university athletes. Front Physiol. (2019) 10(JAN):34. 10.3389/fphys.2019.00034 30761016 PMC6361803

[57] Heard C Willcox M Falvo M Blatt M Helmer D. Effects of linear periodization training on performance gains and injury prevention in a garrisoned military unit. J Mil Veterans Health. (2020) 28(3):23. 33117460 PMC7590922

[58] Stien AN Riiser A Matthew P. Effects of climbing- and resistance-training on climbing-specific performance: a systematic review and meta-analysis. Biol Sport. (2023) 40(1):179–91. 10.5114/biolsport.2023.113295 36636194 PMC9806751

[59] McCrary JM Ackermann BJ Halaki M. A systematic review of the effects of upper body warm-up on performance and injury. Br J Sports Med. (2015) 49(14):935–42. 10.1136/bjsports-2014-094228 25694615

[60] Kay AD Blazevich AJ. Effect of acute static stretch on maximal muscle performance: a systematic review. Med Sci Sports Exerc. (2012) 44(1):154–64. 10.1249/MSS.0b013e318225cb27 21659901

[61] Zech A Hollander K Junge A Steib S Groll A Heiner J Sex differences in injury rates in team-sport athletes: a systematic review and meta-regression analysis. J Sport Heal Sci. (2022) 11(1):104–14. 10.1016/j.jshs.2021.04.003 PMC8847930 34052518

[62] Fulton J Wright K Kelly M Zebrosky B Zanis M Drvol C Injury risk is altered by previous injury: a systematic review of the literature and presentation of causative neuromuscular factors. Int J Sports Phys Ther. (2014) 9(5):583. 25328821 PMC4196323

[63] Lum ZC Park L. Rock climbing injuries and time to return to sport in the recreational climber. J Orthop. (2019) 16(4):361–3. 10.1016/j.jor.2019.04.001 31024194 PMC6476799

[64] Schöffl V Schöffl I Flohé S El-Sheikh Y Lutter C Schoffl V Evaluation of a diagnostic-therapeutic algorithm for finger epiphyseal growth plate stress injuries in adolescent climbers. Am J Sports Med. (2022) 50(1):229–37. 10.1177/03635465211056956 34817275

[65] Clemente FM Afonso J Costa J Oliveira R Pino-Ortega J Rico-González M. Relationships between sleep, athletic and match performance, training load, and injuries: a systematic review of soccer players. Healthcare (Basel). (2021) 9(7):808. 10.3390/healthcare9070808 34206948 PMC8305909

[66] Buscemi V Chang WJ Liston MB McAuley JH Schabrun SM. The role of perceived stress and life stressors in the development of chronic musculoskeletal pain disorders: a systematic review. J Pain. (2019) 20(10):1127–39. 10.1016/j.jpain.2019.02.008 30797962

[67] Close GL Baar K Sale C Bermon S. Nutrition for the prevention and treatment of injuries in track and field athletes. Int J Sport Nutr Exerc Metab. (2019) 29(2):189–97. 10.1123/ijsnem.2018-0290 30676133

