# Supplementary material for: Risk factors and injury prevention strategies for overuse injuries in adult climbers: a systematic review
Source: Front Sports Act Living. 2023 Dec 12;5:1269870. doi: 10.3389/fspor.2023.1269870 (PMC10756908; doi:10.3389/fspor.2023.1269870)
Supplement: Supplementary file 1 [file Datasheet1.docx]

**Supplemental file 1 – PRISMA Checklist**

| **Section and Topic** | **Item #** | **Checklist item** | **Location where item is reported** |
| --- | --- | --- | --- |
| **TITLE** | | |  |
| Title | 1 | Identify the report as a systematic review. | ✔ |
| **ABSTRACT** | | |  |
| Abstract | 2 | See the PRISMA 2020 for Abstracts checklist. | ✔ |
| **INTRODUCTION** | | |  |
| Rationale | 3 | Describe the rationale for the review in the context of existing knowledge. | ✔ |
| Objectives | 4 | Provide an explicit statement of the objective(s) or question(s) the review addresses. | ✔ |
| **METHODS** | | |  |
| Eligibility criteria | 5 | Specify the inclusion and exclusion criteria for the review and how studies were grouped for the syntheses. | ✔ |
| Information sources | 6 | Specify all databases, registers, websites, organisations, reference lists and other sources searched or consulted to identify studies. Specify the date when each source was last searched or consulted. | ✔ |
| Search strategy | 7 | Present the full search strategies for all databases, registers and websites, including any filters and limits used. | ✔ |
| Selection process | 8 | Specify the methods used to decide whether a study met the inclusion criteria of the review, including how many reviewers screened each record and each report retrieved, whether they worked independently, and if applicable, details of automation tools used in the process. | ✔ |
| Data collection process | 9 | Specify the methods used to collect data from reports, including how many reviewers collected data from each report, whether they worked independently, any processes for obtaining or confirming data from study investigators, and if applicable, details of automation tools used in the process. | ✔ |
| Data items | 10a | List and define all outcomes for which data were sought. Specify whether all results that were compatible with each outcome domain in each study were sought (e.g. for all measures, time points, analyses), and if not, the methods used to decide which results to collect. | ✔ |
|  | 10b | List and define all other variables for which data were sought (e.g. participant and intervention characteristics, funding sources). Describe any assumptions made about any missing or unclear information. | ✔ |
| Study risk of bias assessment | 11 | Specify the methods used to assess risk of bias in the included studies, including details of the tool(s) used, how many reviewers assessed each study and whether they worked independently, and if applicable, details of automation tools used in the process. | ✔ |
| Effect measures | 12 | Specify for each outcome the effect measure(s) (e.g. risk ratio, mean difference) used in the synthesis or presentation of results. | ✔ |
| Synthesis methods | 13a | Describe the processes used to decide which studies were eligible for each synthesis (e.g. tabulating the study intervention characteristics and comparing against the planned groups for each synthesis (item #5)). | ✔ |
|  | 13b | Describe any methods required to prepare the data for presentation or synthesis, such as handling of missing summary statistics, or data conversions. | ✔ |
|  | 13c | Describe any methods used to tabulate or visually display results of individual studies and syntheses. | ✔ |
|  | 13d | Describe any methods used to synthesize results and provide a rationale for the choice(s). If meta-analysis was performed, describe the model(s), method(s) to identify the presence and extent of statistical heterogeneity, and software package(s) used. | ✔ |
|  | 13e | Describe any methods used to explore possible causes of heterogeneity among study results (e.g. subgroup analysis, meta-regression). | ✔ |
|  | 13f | Describe any sensitivity analyses conducted to assess robustness of the synthesized results. | ✔ |
| Reporting bias assessment | 14 | Describe any methods used to assess risk of bias due to missing results in a synthesis (arising from reporting biases). | ✔ |
| Certainty assessment | 15 | Describe any methods used to assess certainty (or confidence) in the body of evidence for an outcome. | ✔ |
| **RESULTS** | | |  |
| Study selection | 16a | Describe the results of the search and selection process, from the number of records identified in the search to the number of studies included in the review, ideally using a flow diagram. | ✔ |
|  | 16b | Cite studies that might appear to meet the inclusion criteria, but which were excluded, and explain why they were excluded. | ✔ |
| Study characteristics | 17 | Cite each included study and present its characteristics. | ✔ |
| Risk of bias in studies | 18 | Present assessments of risk of bias for each included study. | ✔ |
| Results of individual studies | 19 | For all outcomes, present, for each study: (a) summary statistics for each group (where appropriate) and (b) an effect estimate and its precision (e.g. confidence/credible interval), ideally using structured tables or plots. | ✔ |
| Results of syntheses | 20a | For each synthesis, briefly summarise the characteristics and risk of bias among contributing studies. | ✔ |
|  | 20b | Present results of all statistical syntheses conducted. If meta-analysis was done, present for each the summary estimate and its precision (e.g. confidence/credible interval) and measures of statistical heterogeneity. If comparing groups, describe the direction of the effect. | ✔ |
|  | 20c | Present results of all investigations of possible causes of heterogeneity among study results. | ✔ |
|  | 20d | Present results of all sensitivity analyses conducted to assess the robustness of the synthesized results. | ✔ |
| Reporting biases | 21 | Present assessments of risk of bias due to missing results (arising from reporting biases) for each synthesis assessed. | ✔ |
| Certainty of evidence | 22 | Present assessments of certainty (or confidence) in the body of evidence for each outcome assessed. | ✔ |
| **DISCUSSION** | | |  |
| Discussion | 23a | Provide a general interpretation of the results in the context of other evidence. | ✔ |
|  | 23b | Discuss any limitations of the evidence included in the review. | ✔ |
|  | 23c | Discuss any limitations of the review processes used. | ✔ |
|  | 23d | Discuss implications of the results for practice, policy, and future research. | ✔ |
| **OTHER INFORMATION** | | |  |
| Registration and protocol | 24a | Provide registration information for the review, including register name and registration number, or state that the review was not registered. | ✔ |
|  | 24b | Indicate where the review protocol can be accessed, or state that a protocol was not prepared. | ✔ |
|  | 24c | Describe and explain any amendments to information provided at registration or in the protocol. | ✔ |
| Support | 25 | Describe sources of financial or non-financial support for the review, and the role of the funders or sponsors in the review. | ✔ |
| Competing interests | 26 | Declare any competing interests of review authors. | ✔ |
| Availability of data, code and other materials | 27 | Report which of the following are publicly available and where they can be found: template data collection forms; data extracted from included studies; data used for all analyses; analytic code; any other materials used in the review. | ✔ |

**Supplemental file 2 – Search strings for Web of Science and Cochrane**

| Search string - Web of Science |
| --- |
| **ALL=(("Climb*" OR "Boulder*" OR "Mountaineering") AND ("Wounds and injuries" OR "Athletic Injuries" OR "Injur*" OR "Overuse") AND ("Risk Factors" OR "Protective Factors" OR "Prevention" OR "Injury Prevention" OR "Prevention Program" OR "Train*")) NOT ALL=(Animals)** |

Cochrane search string


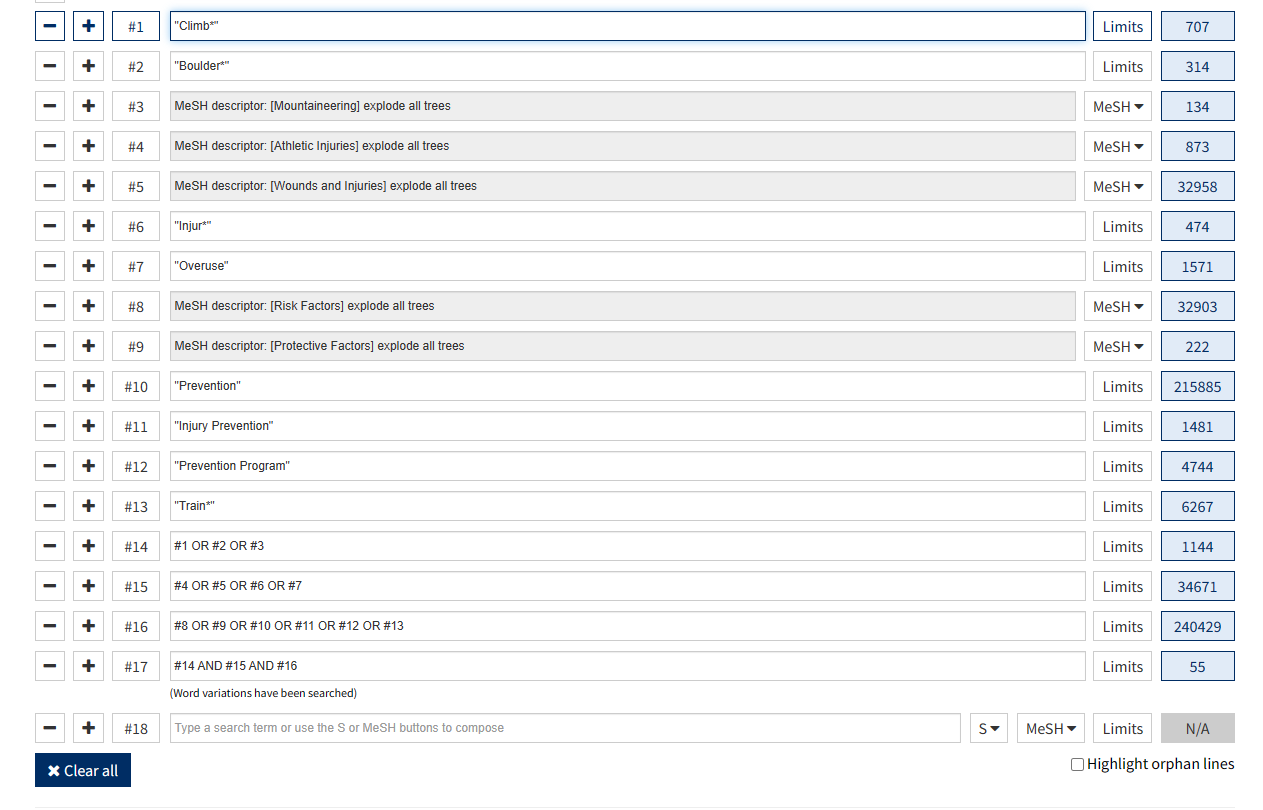


**Supplemental file 3 – Extracted data from the included studies**

| **Study (Year) & Study Type** | **Sample characteristics  Group and number/male-female/age(years)/mass(kg), height(cm), BMI (kg/m²) Climbing type/ climbing level/ training exposure/ years of climbing** | **Injury definition and  incidence/ prevalence** | **Types of overuse injuries  identified** | **Potential risk factors or injury prevention strategies studied** | **Results of risk factors  associated with overuse injury or injury prevention strategies  that reduced injury with  corresponding statistical findings** |
| --- | --- | --- | --- | --- | --- |
| Auer et al. (2021) Prospective (12 months) Explorative cohort study | Adult boulderers, Total n=506, male n=285, female n=221/ male 56% female 44%/ total 30±8years, male 31±8years, female 29±7years/ total 69.0±10.8kg;male 75.2±10.7kg; female 61.0±7.2kg, total 175±9cm; male 181±7cm; female 169±6cm, total 22.4±2.7kg/m²; male 23.1±2.9kg/m²; female 21.4±2.2kg/m² Bouldering/ NA/ 58% 2 to 5hours per week/ 22% more than 5years, 23 less than 1years | 3 possibilities: 1) occurrence of an injury, 2) no occurrence of an injury, and 3) still injured. Injuries were only included in this study if they led to an absence from work or training for more than 24 h; body location, severity and cause of injury were evaluated A considerable proportion of boulderers (44%) reported at least 1 injury during the assessed 12 mo. Injury without acute trauma was sustained by 15% of study population during the study period. Most injuries classified as overuse, and most affected the upper extremities. | Defined just by no acute trauma • Head and neck 0.3% • Upper limbs 21% • Trunk 1% • Lower limbs 3% | • Age • Body weight • Body mass index • Warm up • Taping • Fingerboard training • Years of climbing • Injury history | • No significant difference in age (P=0.48; OR 0.99; 95% CI 0.97–1.02), size (P=0.88; OR 1.00; 95% CI 0.98–1.02), body weight (P=0.85; OR 1.01; 95% CI 0.99–1.02), or body mass index (P=0.88; OR 1.01; 95% CI 0.93–1.08) between injured and non-injured climbers. • Preventive measures or specific training for climbing no protective effect. • Warming up fingers, taping locations other than the fingers, and using a fingerboard increased the risk of injury. In secondary analysis considering confounders, only warming up the fingers still had an effect.  • No significant difference in injury rate based on warming up duration (P=0.97; OR 1.00; 95% CI 0.90–1.12). **•** Bouldering experience did not affect general injury rate (P=0.40; OR 1.25; 95% CI 0.74–2.25) or rate of injury UIAA ≥2 (P=0.59; OR 1.04; 95% CI 0.90–1.22). • Participants with less than 1 year of bouldering experience were more often affected by lower extremity injuries (n=507; P=0.027; OR 1.85; 95% CI 1.07–3.21). • Participants with a history of injury had a higher risk of sustaining another injury (P=0.020; OR 1.54; 95% CI 1.07–2.21). |
| Backe et al., (2009) Cross-sectional study | Youth and adult climbers, members of  Swedish Climbing Association, n=355/ mean age=30 years (range: 9-67 years)/ NA, NA, <18.5kg/m²=8.8%, 18.5-24.9kg/m²=83.3%, >25.0kg/m²=7.9% Sport climbers=42.4%, Trad climbers=31.4%, Boulderers=23.1%, Ice/Alpine climbers= 3.1%/ NA/ Total recorded exposure time to climbing=49986 hours/ 0-4years=42.4%, 5-9years=25.4%, 10+years=21.4% | Both overuse and traumatic injuries  included based on retrospective  self-report. Traumatic defined as acute  onset, overuse as repeat microtrauma. Injuries defined as climbing related  and resulting in an injury treatment intervention. 208 injuries reported in  total, 4.2 injuries per 1000 hours of  climbing. 93% classified as  overuse. | Injuries defined by anatomical location:  hand/finger/wrist=42%, upper arm/shoulder=39%, lower limb=13%, head/neck=3%, trunk=2% | • Time exposed to climbing per year • Body mass index (BMI) • Sex • Age group  • Type of climbing practiced (trad/sport/bouldering) • Years of climbing experience | Primary risk factor analysis for climbing injury: • Increased BMI (p<0.015) • Bouldering (p<0.047) Risk factor analysis for re-injury: • Time climbing per year (p=0.439) • BMI (p=0.121) • Sex (male) (p=0.019) • Age group (20-45 vs. <20 yrs.) (p=0.003) • Age group (46+ vs. <20 yrs.) (p<0.001) • Bouldering (p=0.122) • Sport climbing (p=0.719) • Years climbing experience (5-9 vs. 0–4 yrs.) (p=0.775) • Years climbing experience (10+ vs. 0–4 yrs.) (p=0.060) |
| Beeler et al. (2021)  Retrospective cohort study | Adult male high level rock climbers, n=31/ only male/ 47.1±6.07years/ 72.8±6.68kg, 178±6cm, BMI=23±1.59 Rock climbing/ IRCRA>18/ 12.3hours per week/ mean=31.6 years | All climbers were asked about shoulder pain in the past (lifetime), in the 6 months before clinical examination, and on the day of clinical examination (point time). all climbers were asked about acute or chronic shoulder injuries and the need for treatment. Acute injuries were defined as a single trauma, with a sudden onset of symptoms. Chronic injuries were defined as overstrain injuries, with no sudden onset of symptoms. Of the 31 climbers, 22 (71%) had shoulder pain for an average of 3.1 years (range 1⁄4 1 month to 25 years), 17 (55%) reported pain in one or both shoulders in the 6 months before examination, and 8 (26%) reported shoulder pain on the day of examination. Twelve of the 31 (39%) had an acute injury and 5 (16%) had a chronic injury, which were directly associated with climbing | • 2 rotator cuff tears [RCTs] • 5 muscle strains • 1 AC joint dislocation • 2 AC joint osteoarthritis • 1 glenohumeral osteoarthriti • Labral tears • Biceps tendon abnormalities • Cartilage lesions • Osteoarthritic changes | • Dominant vs non dominant shoulder • Climbing grade | • No statistical differences between pathologic findings of the dominant and nondominant shoulders. • Highest climbing grade showed significant positive correlations with long biceps tendon tears (r 1⁄4 0.242), superior labral tears (r 1⁄4 0.34), anterior labral tears (r 1⁄4 0.32), posterior labral tears (r 1⁄4 0.22), sublabral cysts (r 1⁄4 0.221), cartilage defects (r 1⁄4 0.41), and joint effusion (r 1⁄4 0.311).  Prolonged rock climbing at a high level is associated with a high prevalence of degenerative changes to the glenohumeral joint as seen on MRI scans. • Changes correlated with the intensity of climbing. Prolonged rock climbing at a high level is associated with a high prevalence of shoulder pain and degenerative changes in the glenohumeral joint. Labral tears, cartilage lesions, and biceps tendon pathologies are frequently observed. |
| Bollen et al., (1988) Cross-sectional study | Adult and youth climbers, n=86 / 10 female, 76 male / mean age 27 (15-46) years / NA, NA, NA  Rock climbing / difficulty "evenly distribtuted amoung the 'extreme grades'" / NA / NA | As reported by the questionaire, questionaire not included | 20 elbow injuries with 10 related to climbers elbow, 67 hand injuries with 43 possibly related to climbers finger; of the finger overuse 42% were ring and 53% were middle finger | • Repetitive motions, crimp grip | "Climbers elbow": associated with repetitive pull-ups or traversing on climbing walls; "climbers finger" associated with crimp grip used by most climbers |
| Buda et al., (2013) Cross-sectional study | Adult competitive and recreational climbers,  n= 211 / 81.9% male, 18.1% female/ mean age= 31.7 years/ NA, NA, 21.9kg/m² Indoor= 29.2%, Outdoor= 84.7%, Boulder= 61.1%, Mountaineering= 13.9%/ IRCRA grade 7-28/ mean 8.6 training sessions per month/ mean experience 7.6 years per month | Overuse injuries of the foot, based  upon clinical signs and symptoms. No incidence or prevalence reported. | Nail disease (65.3%), recurrent ankle sprains (27.8%), retrocalcaneal bursitis (19.4%), Achilles tendinitis (12.5%), metatarsalgia (12.5%), and plantar fasciitis (5.6%). | • Sex • Shoe type • Level of climbing • Foot type • Toe deformities • Body weight • Years of experience • Monthly training sessions | • Male sex (30.5% of males with recurrent ankle sprains, vs. 15.4% of females) • The use of high-type shoes (16.7% vs. 4.3% with metatarsalgia) • High degree of climbing difficulty • High competitive level associated with various foot conditions (p= 0.009 to 0.07) • Years experience associated with nail disease (p = 0.09) • Higher monthly training sessions associated with recurrent ankle sprains (10 vs. 8.1 sessions; p = 0.13) |
| Carmeli et al., (2002) Cross-sectional study | Adolescent and young climbers in Tel Aviv, Israel, n=37 (19 adolescent climbers/ 18 young climbers/ 67%male, 33% female/ adolescent climbers mean age=14years, young climbers mean age=24years, total range=9 to 34years/ NA, NA, NA Sport climbing/ nonprofessional (recreational)/ NA/ NA | Self-reported soft tissue injury to the hands or fingers, assessed via questionnaire.  Incidence unavailable. | Injuries were classified via a functional diagnsoes, for example related to the ligaments or tendons of the fingers/hands. | • Sex  • Age (19-34 vs. 9-18 yrs.) • Training frequency • Grip strength  • Demographics • Climbing profile • Past injuries | • Sex (male) (p<0.05) • Age (19-34 vs. 9-18 yrs.) (p<0.05) • Training frequency (practicing 4-5x per week reported more diverse wrist and finger injuries) ( p<0.05) • Grip strength: “mild to moderate correlation": (r=0.26 and r=0.41) (p<0.05) |
| Cobos-Moreno et al., (2022),  Cross-sectional study | Adult and youth climbers, n=43 / 32 male 21 female / 27.5±12.83years / 57.75±16.94kg / 162±17cm, NA  Sport climbing on a climbing wall, 60% also pratice rock climbing / NA / 6.6h per week (range: 3-15) / 7years (range: 2-22) | Morphostructural alterations of the foot (hallux valgus, hallux limitus, claw toes), dermatological alteration (blisters, nail problems, hyperkeratotic patterns, hematomas); frequency of foot injuries 73.59% (41.51% male, 32.08% female) | Damaged joint (claw and mallet in tow) , dermic injury, digital injury, nail injury | • Climbing time, years of climbing  • Degree of difficulty  • Smaller shoes | • Foot injury: 38.48% climbing<7years; 61.5% climbing≥7years: bursitis (skin lesion) of first toe more prevalent in people with smaller shoes and higher degree of climbing difficulty  • Serious nail alterations associated with degree of climbing difficulty; years of climbing practice associated with presence of injury (p=0.035) |
| Gerdes et al., (2006) Cross-sectional study | Youth and adult rock climbers, n=1887/ 87% male, 13% female/ mean age=29 years (range: 10 to 66 years)/ NA, NA, NA Bouldering, top rope, trad, lead, indoor and outdoors, climbing competitons (16.6%)/ IRCRA grade 7-9 (35%), 5.10-5.11 (49%)/ climb >10 days per year (98%), >50 days per year (69%)/ mean years experience= 7.5 years (range: 0 to 48 years) | Subjects asked to identify their three most significant climbing injuries. Injury data collected included body part, type of injury, type of climbing during injury, medical care sought, and recovery time. "The mean number of injuries documented among the subjects was 2.33 (SE 0.14), with 17.9% of participants reporting no history of climbing injuries." Total of 2,472 injuries reported, indicating career incidence proportion of 131 injuries/100 participants. | "The most common body parts injured were: fingers (27.5%), ankle (12.6%), elbow (9.2%), and shoulder (9.2%). Upper extremity injuries represented 57.6% of all injuries. Sprain/strain was the most common injury type (39.1%), followed by chronic overuse (19.8%)." "Sprains and strains were most common in fingers (31.9%) and ankle (23.0%). Chronic overuse injuries occurred most often in the upper extremities: finger (33.9%) and elbow (28.8%)." | • Climbing discipline • Indoor vs. Outdoor climbing • Familiar or unfamiliar climbing environment • Alcohol and drug use | • Climbing discipline: more injuries in traditional climbing (mean 2.53 vs. 1.92; p<0.001) or free solo climbing (mean 3.30 vs. 2.09; p<0.001) (likely related to traumatic injury) • Indoors vs. outdoors: 47.7% and 52.3% of injuries  • More injuries occrued in a familiar location versus an unfamiliar/novel area: (79.2% and 20.8%) o 4.3% (95% Confidence Interval [CI]; 3.6, 5.2) injuries occured in ‘beginner’ climbers o 28.3% (95% CI; 26.6, 30.1) by ‘intermediate’ group o 46.3% (95% CI; 44.4, 48.3) by ‘advanced’ group o 21.2% (95% CI; 19.6, 22.9) by ‘expert’ group • Use of alcohol or drugs associated with injury risk (p<0.008) |
| Grønhaug (2018) Retrospective survey | Active climbers, n= 667/ 72.1% male, 27.9% female/ 58.7% were 26–40 years of age (28.3%: 26–30 years and 30.4%: 31–40 years)/ NA, NA, NA Boulderers and route climbers/ IRCRA grade 14-18 (49%), 19-25 (34%)/ NA, elite (3.5%), international elite (0.2%)/ 3–5 years (30.1%), 6–10 years (22.3%), 11+ years (29.1%) | Self-reported injury via web-based questionnaire. Defined as "climbing-related chronic injury" for at least 6 months, with follow-up question regarding anatomical location. "Of the 667 respondents, 385 reported to have sustained an injury during the last 6 months (58%). There were 289 males and 96 females among the ones who reported an injury (75% and 25% of the injured, respectively)." | "The three most frequent sites of injury were fingers (41.3%), shoulders (19.5%) and elbows (17.7%). The most frequent injuries for the females were fingers (29.2%), shoulder (21.9%), wrist (12.5%), elbow (11.5%) and foot/ankle (10.4%). The most frequent injuries for the males were fingers (45.3%), elbow (19.7%) and shoulder (18.7%)." | • Gender • Type of climbing • Experience level | • Gender: Male risk factor for finger and elbow injuries (fingers - 45.3% of the males and 29.2% of the females; elbows - 19.7% of the males and 11.5% of the females • Gender: Female risk factor for ankle injuries (3.8% of the males and 10.4% of the females) • Type of climbing: higher prevalence of injuries outdoors vs. indoors, especially in male outdoor climbers (74% injury prevalence) • Experience level: Higher prevalence of injuries in male climbers with higher bouldering grades achieved (international elite 67% vs. recrational 57%). Also partly reflected in female climbers, although recreational female climbers have higher prevelance (55%) than intermediate (43%). |
| Grønhaug (2019) Cross-sectional study | Adult climbers, n= 667/ 72% male, 28% female/ 26-40 years old/ NA, NA, 22.78kg/m² Boulders, trad+sport climbing, ice climbing/ IRCRA grade range 7-29 (51% grade 14-18)/ range <1 - >10 hours per week (75% 4-10 hours per week)/ 3–5 years 30.1%, 6–10 years 22.3%, 11+ years 29.1% | Climbing related chronic injuries within  the last 6 months. Incidence proportion of 58 injuries/100 participants in last 6 months. | Chronic injuries identified by anatomical  location only. Most common sites fingers (41%), shoulders (19.5%), elbow (18%), but also toe, foot, ankle, calf, knee, thigh, wrist, hip, head | • BMI | "The findings in this study indicate that there are no associations between level of climbing, chronic  injuries or training volume and BMI. It is also found that the average BMI is similar across groups  regardless of genderand level of performance." P=0.44, R²=0.001 |
| Jones et al., (2015) Retrospective cohort study | Actively engaged in rock climbing over the previous 12 months, n=201/ Male n=163; female n=38/ male 35.2±11.8years; female 35.1±10.7years/ NA, NA, NA Rock climbing/ NA/ NA/ male=13.9+11.8years; female=11.6+9.2years | Classified previous injury as resulting from fall, repetitive overuse, or strenuous move. Participants were categorized as reinjured if they reported the same injury site and cause at least twice within 12 months. 101 particioants sustained injury in least 12 months. Of these 101 participants, 36 sustained at least one reinjury. The total number of reinjuries in these 36 participants was 82, with a clinical incidence of 1.78 reinjuries per climber. The epidemiological IP for reinjury was 0.356 (95% CI 0.347 to 0.368). Thus, the average probability of sustaining at least one reinjury as a result of climbing was 35.6% (95% CI 34.71% to 36.8%). | • Repetitive overuse  • Fingers most common site 26% • Frequency (participants):Shoulder (5), Elbow (6), other (7), wrist (1), forearm (1) | • Solo grade • Outdoor sport lead grade • Indoor sport lead grade • Bouldering frequency • Bouldering grade • Previous injury | • 29 participants sustained at least one reinjury as a result of repetitive overuse. • Participants who had sustained a previous injury were more likely to sustain a reinjury (p<0.001) with the relative risk of reinjury being 1.55 (95% CI 1.34 to 1.80) • The IP for reinjury for repetitive overuse was 0.630 (95% CI 0.49 to 0.77);  • The average probability of sustaining at least one reinjury as a result of repetitive overuse was 63.0% (95% CI 49.0% to 77.0%); the average probability of sustaining at least one injury as a result of strenuous overuse was 30.4% (95% CI 17.1% to 43.7%). • Reinjuries sustained through repetitive overuse accounted for 80.5% of total reinjuries and was positively associated with solo grade, bouldering grade, bouldering frequency, outdoor sport lead grade and indoor sport lead grade. Individuals who performed a high standard were more likely to sustain a repetitive overuse reinjury. |
| Jones et al., (2008) Cross-sectional study | Adult climbers from indoor and outdoor climbing venues in the UK, n=201/ 81% male, 19% female/ mean age=35 years (range: 16-62)/ NA, NA, NA Indoor and outdoor rock climbers/ NA/ NA/ Mean of 12 years climbing experience | Anatomical site of injury that occurred within the last 12 months. Injuriy defined as requiring medical attention or reulting in withdrawal of climbing participation for >1 day. Injuries categorised into overuse, overstrain and fall. 12 month incidence proportion of 137 injuries/100 participants. | "101 climbers (50%) had sustained at least 1 injury in the past 12 months, causing a total of 275 distinct anatomical injuries. Of these, 21 climbers (10%) had sustained acute climbing injuries as a result of a fall, 67 (33%) had sustained chronic overuse injuries, and 57 (28%) had sustained acute injuries caused by strenuous climbing moves." "Subcutaneous trauma to the finger and shoulder were the most common injuries resulting from both overuse and strenuous moves. Elbow, forearm, wrist and other injuries were also reported." | • Age • Sex • Climbing experience • Soloing frequency • Soloing grade • Traditional lead frequency • Traditional lead grade • Sport lead frequency • Sport lead grade • Indoor lead frequency • Indoor lead grade • Bouldering frequency • Bouldering grade | • Age (p>0.05) • Sex (male) (p>0.05) • Years climbing experience (p>0.05) • Soloing frequency for overuse injury (p<0.05) (OR=1.79, 95% CI; 1.14, 2.83) • Soloing grade (p>0.05) • Traditional lead frequency (p>0.05) • Traditional lead grade for overuse injury (p<0.05) (OR=1.25, 95% CI; 1.07, 1.46) • Sport lead frequency for overuse injury (p<0.05) (OR=1.49, 95% CI; 1.05, 2.13) • Sport lead grade for overuse injury (p<0.05) (OR=1.28, 95% CI; 1.05, 1.56) • Indoor lead frequency for overuse injury (p<0.05) (OR=1.21, 95% CI; 1.03, 1.42) • Indoor lead grade for overuse injury (p<0.05) (OR=1.42, 95% CI; 1.17, 1.71) • Bouldering frequency for overuse injury (p<0.05) (OR=1.24, 95% CI; 1.07, 1.43) • Bouldering grade for overuse injury (p<0.05) (OR=1.42, 95% CI; 1.16, 1.73) and strenuous moves (p<0.05) (OR=1.24, 95% CI; 1.02, 1.50) |
| Josephson et al., (2007) Cross-sectional plus prospective cohort (12 months) | Adult boulderers from 12 popular bouldering destinations is North America, n=152, outdoors=109, indoors=43/ male = outdoors: 56.9%, indoors: 67.4%/ mean age= outdoors: 24.7±5.1years, indoors: 27.5±6.5years/ female; outdoors =56.6±8.4kg, indoors =57.2±8.4kg, male; outdoors =71.4±9.4kg, indoors =76.3±11.0kg/ NA/ female: outdoors = 21.1±2.6kg/m², indoors = 27.5±4.6kg/m²; male: outdoors = 22.4±2.1kg/m², indoors = 28.4±6.8kg/m² Bouldering only/ IRCRA grade 18 to 20/ NA/ mean years experience 3.9 to 4.4 | Injury defined by body part and simple "yes/no".  Incidence proportion of injuries outdoors= 103 inuries per 100 participants/year Incidence proportion of injuries indoors= 127 inuries per 100 participants/year | For the outdoor cohort, 61% of injuries were in the fingers, 26% in the shoulder, and 26% in the elbow. For the indoor cohort, 27% of injuries were in the fingers, 36% in the shoulder, and 27% in the elbow. Lower extremity injuries were more associated with falls (outside the scope of this review). | Risk factors: • Outdoor vs. indoor bouldering • Previous history of finger injury • Sex • Years climbing experience • BMI • Weight • Climbing ability • BMI • Weight Prevention measures: • Warm-up • Stretching • Regular yoga practice • Finger taping • Wrist taping • Glucosamine • Other supplement use • Heating hands prior to climbing • Taking time off to prevent injuries • Use of corticosteroid injections • Weight training • Presence of spotters • Use of bouldering pads | Risk factor examination: For finger injuries (non-tramuatic): • Outdoor vs. indoor bouldering: higher outdoor (19 (61%) vs. 6 (27%); 95% CI for risk difference: -10, -3) • Previous history of finger injury (p=0.03) (OR=4.0, 95% CI; 1.2, 13.6) • Sex (p>0.05) • Years climbing experience (p>0.05) • BMI (p>0.05) • Weight (p>0.05) • Climbing ability (p>0.05) Prevention measure examination: • Warm-up (p>0.05) • Stretching (p>0.05) • Regular yoga practice (p>0.05) • Weight training and wrist taping offers protective effect |
| Killian et al., (1998) Retrospective survey | Adult climbers from five climbing locations in the USA, n= 100/ 73% male, 23% female/ mean age: 25.6 years (range 14 to 45)/ NA, NA, NA Presumed route climbers (outdoors)/ mean IRCRA grade = 14/ NA/ NA | Self-report questionnaire, specifically asking about discomfort and symptoms in the feet associated with climbing. 81% point prevalence of pain/discomfort in the feet. | • Foot pain or discomfort (81%) • Numbness in feet (30%) • Tingling in feet (41.5%) | • Shoe size • Climbing level | • No association between shoe size or climbing level, and pain/discomfort in the foot • A significant correlation between climbing ability and extent of shoe-size reduction was found (ρ = .28; P < .01) • Strong correlation found between shoe-size reduction and tingling (ρ = .35; P < .001) in the foot, and climbing level and tingling in foot (ρ = .23; P < .05)  • No correlation found between shoe-size reduction or skill level and foot numbness |
| Kozin et al., (2021) Randomized Controlled Trial Injury Prevention Intervention Study | Amateur male student rock climbers in Ukraine, n=84 (intervention group=40, control group=44)/ 100% males/ 18-19years/ Control group: 172.5±8.5 cm, 65.2 ± 6.5 kg; intervention group: 173.4±8.7 cm, 66.1±6.6 kg; BMI NA Rock climbing (probably sport climbers)/ NA/ NA/ control mean years experience=2.4±1.2 years; intervention mean years experience=2.2±1.4 years | Injuries recorded for one year follow-up post intervention: "The following injuries were registered: shoulder injuries in severity: minor, moderate, severe. Minor injuries included those that healed in less than 1 month, medium injuries to those that healed in 2–3 months, and complex injuries to those that healed within 6–12 months. Injuries were recorded independently by athletes, coaches and teachers. The severity of injuries was determined by the time required for complete recovery." "Injury rate per 1000 athlete exposures of all recorded shoulder injuries in the control group during 1 year of the experiment was 3.182 (95% CI, 1.061; 5.367), it was in the intervention group 0.5 (95% CI, 0.068; 1.375), P < 0.001." | Shoulder injuries classified by severity, but not explicitly by pathology. | Tailored injury prevention program (1-yer), designed to prevent shoulder injuries. Exercises included "closed kinematic chain exercises, with eccentric and strength exercises". Exercises were push-ups on rings, TRX rows, crossbar pull-ups, eccentric wrist pronation. Exercises were performed 3-4 times per week, 15 minutes allocated per session. Repetitions performed 5-20 times, depending on type of exercise and capacoty of individual participant. | Positive effect of injury prevntion program in reducing shoulder injuries was reported: "The total number of all recorded shoulder injuries during 1 year of the experiment was 21 in the control group and 3 in the experimental group. The number of athlete exposures during 1 year of the experiment was 6600 in the control group and 6000 in the intervention group. Injury rate per 1000 athlete exposures of all recorded shoulder injuries in the control group during 1 year of the experiment was 3.182 (95% CI, 1.061; 5.367), it was in the intervention group 0.5 (95% CI, 0.068; 1.375), P < 0.001." |
| Kozin et al., (2021a) Randomized Controlled Trial Injury Prevention Intervention Study | Amateur male student rock climbers in Ukraine, n=84 (intervention group=40, control group=44)/ 100% males/ 18-19years/ Control group: 172.5±8.5 cm, 65.2 ± 6.5 kg; intervention group: 173.4±8.7 cm, 66.1±6.6 kg; BMI NA Rock climbing (probably sport climbers)/ NA/ NA/ control mean years experience=2.4±1.2 years; intervention mean years experience=2.2±1.4 years | Injuries recorded for one year follow-up post intervention. Elbow injuries were recorded, without a clear definition. Elbow injuries were categorized as "mild", "moderate", or "severe". "The total number of all recorded elbow injuries during 1 year of the experiment was 29 in the control group and 5 in the intervention group." "The risk (Injury rate) of elbow injuries Mild Degree per 1000 athlete exposures during 1 year of the experiment in the control group was 1.82 (95% CI, 1.071; 2.403), in the intervention group – 0.5 (95% CI, 0.064; 0.979)." | Elbow injuries classified by severity, but not explicitly by pathology. | • Tailored injury prevention program (1-year), designed to prevent injuries. Exercises included "closed kinematic chain exercises, with eccentric and strength exercises". Exercises were push-ups on rings, TRX rows, crossbar pull-ups, eccentric wrist pronation. Exercises were performed 3-4 times per week, 15 minutes allocated per session. Repetitions performed 5-20 times, depending on type of exercise and capacoty of individual participant. • Improvements in one-arm hang climbing technique were measured pre and post, determined via subjective "expert criteria" and biomechanical video analysis. | Risk of elbow injuriy was reduced in the training intervention group. "The chance of getting mild elbow injuries in the control group was 4.625 times higher than in the intervention group (95% CI: 1.198; 17.854). The chance of getting moderate elbow injuries in the control group was 5.588 times higher than in the intervention group (95% CI: 1.143; 27.324)." "There was a significant improvement in the results of biomechanical analysis of the hang technique in rock climbing among athletes of the intervention group: after the experiment, the angle between the shoulder and the clavicle decreased significantly in the athletes of the intervention group (p <0.001), while in the control group these changes were not significant (p> 0.05). A high reliability of the influence of the level of technique proficiency on the number of injuries was found (p <0.001). |
| Lion et al. (2016) Cross-sectional retrospective | Total of n=528/ male n=439 (83.1%) female n=89 (16.9%)/ 29.2±9.5years/ NA, NA, <19.9kg/m² n=109 (20.6%); 20-20.9kg/m² n=120 (22.7%); 21-22kg/m².49 n=156 (29.5%); >22.5kg/m² n=143 (27.1%) Natural n=201 (38.1%); Artificial n=327 (61.9%)/ IRCRA grade 12-20/ 2 sessions per week n=156 (29.5%); 3 sessions per week n=240 (45.4%); >3 sessions per week n=132 (25%)/ at least 3 years climbing experience. | All complaints concerning traumatic lesions of the following four anatomic structures of the hand (including fingers): tendons (e.g., tendonitis, tenosynovitis), pulleys, muscles, and joints (e.g., sprain). These health disorders of the locomotor system are called injuries hereafter, irrespective of the severity (i.e., time-loss injuries). It was acknowledged that climbers did not report Climbing related injuries of the hand (CRIH) resulting from falls. The majority of climbers reported at least one CRIH (n = 356, 67.4%). Mainly observed tendon injuries (n = 328, 92.1% of those who have had CRIH). Conversely, pulley (n = 191, 53.7%), muscle (n = 85, 23.9%), and joint injuries (n = 94, 26.4%) occurred less often. | Tendon injuries, finger pulley injuries, muscle injuries and joint sprains. | • Climbing level • BMI • Hydration • BMI • Warming up • Cooling down • Cardiovascular training | • Climbing proficiency level and BMI significant risk factors for CRIH, higher levels and higher BMIs greater risk. • Tendon injuries more likely with BMIs above 20 kg/m2, pulley injuries more likely with BMIs above 21 kg/m2. • Hydration over 0.5 L/h increases the risk of muscle injuries. • Use of crimp grips associated with pulley injuries, More force on single tendons and retinaculi. • Traditional preventive measures like warming up and cooling down/stretching sessions not effective enough to prevent climbing injuries. |
| Logan et al., (2005) Cross-sectional retrospective survey | Adult climbers, members of Climber's Club Great Britain, n=545/ 91.4% male, 8.6% female/ mean age 54 years (range from 23 to 93 years)/ NA, NA, NA Unknown, likely wide range of climbing disciplines/ NA/ lifetime climbing intesnity score (climbing exposure) was 2071 and 2635 in each group/ NA | Primary outcome of Dupuytren's disease, self-diagnosed via information and illustrations. Other injuries assessed included previous wrist or hand injury, including fractures, dislocations, crush injuries, burns, cuts, abrasions, and tendon damage.  Dupuytren's disease reported with a 19,.5% prevalence in males (97 men). | Data only reported for Dupuytren's disease | • Family history • Blood group  • Manual occupation  • Alcohol consumption • Smoking  • Prescribed treatment for epilepsy • Climbing intensity score | • Positive family history: 16% of climbers with Dupuytren's disease had a positive family history, compared to only 4% of climbers without the disease. This difference was found to be statistically significant (p<0.001). • Age: The mean age of climbers with Dupuytren's disease was 58.4 years, compared to 52.6 years for those without the disease. The severity of the disease was found to be significantly correlated with the age of onset (r=0.241, p=0.017) • Climbing intensity: Climbers with Dupuytren's disease had a significantly higher average climbing intensity score (2635) compared to those without the disease (2071). This difference was statistically significant (although data not reported). |
| Lutter et al. (2018) Prospective follow-up study | Adult high level rock climbers, n=31/ male=23, female=8/ 27±9.6 (15-51)years/ NA, NA, NA Rock climbing/ IRCRA average grade 20/ NA / 10.9±7.1 (2-26)years | Bone marrow edemas (BME) can be divided into the following categories: mechanical BME (chronic stress, fracture, malalignment, and degenerative), ischemic BME (osteonecrosis), inflammatory BME (rheumatoid arthritis and infectious), idiopathic BME (transient BME), or BME due to malignoma. Only patients suffering from load-dependent pain, unable to practice rock climbing, and with discomfort during simple daily tasks were included in the study. In 29 of the 31 investigated athletes, a BME not caused by an incident such as trauma, inflammatory factors, or tumor was found in the distal radius, the distal ulna, or in the carpal bones. Bone marrow edema in combination with, or caused by other underlying conditions was detected in 10 patients (stress-induced fracture, nonunion, osteoarthritis, negative ulna variance, and fractured cyst). | Bone marrow edema, hamate and capitate injuries, diffuse pain in hand and wrist joint, edema in the forearm | • Anatomic predispositions  • High intensity training • Climbing level • Sex • Climbing experience • Age | • Anatomic factors like incongruent wrists or joint instability can lead to wrist problems. • High-intensity training can cause edema. • Hamate and capitate injuries were more common in climbers with higher level (P = 0.001). • 74% of injuries were bone marrow edema (BME) in the wrist and carpal bones, with higher climbing experience (13.2 ± 6.8 years) and level (9.1 ± 1.3 UIAA). • Hamate and capitate injuries had higher climbing level (9.6 ± 0.7 UIAA) and experience (12.1 ± 5.4 years) (P = 0.001). • 10% of athletes had metacarpal bone edema with lower climbing experience (3.7 ± 0.5 years) and competency (7.8 ± 0.4 UIAA). • Patients with forearm edema were significantly younger (17.8 ± 2.5 years) compared to other injuries (P = 0.018). |
| Lutter et al. (2019) Single-centre injury surveillence study (over 3-year period) | Adult rock climbers and boulderers split in 3 age groups All n=198, 35-49years n=146, 50-64years n=48, ≥65years n=4/ All m-f=150-48, 35-49years m-f=104-42, 50-64years m-f=42-6, ≥65years m-f=4-0/ All=44±7years, 35-49years=41±4years, 50-64years=53±3years, ≥65years=72±5years/ All=68.7±9.5kg; 35-49years=67.6±9.4kg; 50-64years=71.9±9.4kg; ≥65years=67.3±5.9kg, All=175±9cm; 35-49years=174±9cm; 50-64years=178±9cm; ≥65years=172±4.6cm/ Rock climbing and bouldering/ Mean IRCRA grade 20/ All=6.6±4.9hours per week, 35-49years=6.7±5.4hours per week, 50-64years=6.6±4.1, ≥65years=1.8±1/ All=19.8±11.2years, 35-49years=17.8±9.5years, 50-64years=24.4±11.6years, ≥65yr=53.4±7years | Acute injuries were defined as injuries with a sudden onset during climbing without any history of symptoms, and overuse injuries were defined as chronic injuries without a singular causing event or specific trauma that developed during or after climbing. Among overuse injuries, degenerative conditions were defined as nonreversible injuries that worsened over time. During the 3-year period, 198 patients were treated with a total number of 275 independent injuries caused by rock climbing or bouldering. Among the 275 injuries, 187 (68%) were overuse injuries and 88 (32%) were acute injuries. | •15% of all injuries, a finger pulley laceration (single or multiple pulley rupture) was the most frequent diagnosis • Finger joint capsulitis (12%) • Subacromial impingement (10%) • Finger tenosynovitis (7%) Degenerative conditions: • Subacromial impingement syndrome was the most frequent diagnosis (32%) • Chronic superior labral tear from anterior to posterior lesions (22%) • Osteoarthritis of the fingers (14%) • Dupuytren disease (10%) | • Age • Sex • Experienced joint displacements or joint fractures • Climbing level • Climbing hours per week • Years of climbing • Height  • Weight | • No significant difference between age groups for inury risk, thugh attributed to small sample size and selection bias • No significant association between climber's climbing level, climbing hours per week, years of climbing, height, weight, and sex and the development of an acute or overuse injury. • With a ratio of 3:1, a significantly higher rate of injured male climbers than female climbers was observed. Climbing levels and climbing experience were both significantly higher in males. • Subacromial impingement syndrome occurred twice as frequently in older athletes (10% of all patients) compared to younger athletes in the existing literature. Osteoarthritis of the finger was present in 4% of the athletes. • Degenerative conditions such as impingement syndrome of the shoulder or osteoarthritis of the fingers are common injuries in older athletes. |
| Nelson et al. (2017) Cross-sectional study | Members of local climbing clubs and gyms  Total n=397/ male n=338 (85%); female n=59 (15%)/ 32.5years mean/ NA, NA, NA Bouldering n=301 (75.81%); Top roping n=309 (77.83%); Lead n=362 (91.19%); Crack n=275 (69.26%); Other(ice, indoor wall, etc) n=229 (57.68%)/ IRCRA grade 1-18 (divided into 3 groups)/ Once a year or once every 3mo n=11 (2.77%); once a month n=36 (9.06%); once a week n=350 (88.16%)/ <1year n=16 (4.03%); 1-2years n=59 (14.86%); 3-4years n=70 (17.65%); 5-6years n=45 (11.33%); 7-8years n=22 (5.54%); >8years n=185 (46.59%) | Associations between demographic covariates and the outcome of whether or not any upper extremity injury was sustained. A total number of 357 (90%) participants reported sustaining an upper extremity injury. Fingers (41%) were the most commonly injured, followed by the shoulder/arm (20%) and the elbow/forearm (19%). Most commonly reported injuries were abrasion/bruises (66%), followed by lacerations (57%) and tendon injuries (56%). Of those reporting a tendon injury, 86% were reported as a flexor tendon or pulley injury of the hand. Fractures were reported only 8% of the time. Of the 357 climbers with upper extremity injuries, 56% reported chronic or residual symptoms with bone and joint pain (ligament instability) being themost common complaint (69%). | Finger injuries, shoulder/upper arm injuries, elbow/forearm injuries, tendon injuries (flexor tendon or pulley injuries). | • Age • Gender • Level of experience • Years of climbing • Frequency of climbing • Grade of climbing • Training for climbing • Use of protective gear | • Climbing frequency: The odds of sustaining an upper extremity injury increased as the frequency of climbing increased (P = 0.038). • Age: no association with injury • Hand and finger injuries: Among those who climb once a week, the odds of sustaining finger injuries were 2.49 times higher (95% CI: 1.27-4.90) compared to those who climb at most once a month. • Climbing level: Semi and professional climbers had a higher incidence of injuries, which were positively related to the amount of hours spent in training. • Sex: Females were twice as likely as males to sustain a shoulder/upper arm injury (OR: 2.05, 95% CI: 1.11-3.77, P = 0.02) after adjusting for climbing frequency and years of climbing. Additionally, among females who sustained a shoulder/upper arm injury, 33% underwent surgery compared with 16% of the males who reported a shoulder/upper arm injury (P = 0.033). • Tendon injuries: 56% of climbers surveyed had tendon injuries, with 86% of these being flexor tendon or pulley injuries. |
| Neuhof et al., (2011) Cross-sectional study | Adult sport climbers, n=1962/ 81% male, 19% female/ mean age: 32.82±9.4 years (range 13–60)/ NA, NA, 22.2±2.0kg/m² Sport climbers/ IRCRA grade 3 to 28/ Summer months (April until September): 102±7.4hours, winter months (October until March): 3.7±3.9hours/ years experience: 10.0±7.5 years | "A climbing injury was defined as an injury that occurred during a sport climbing activity that resulted in a professional medical treatment intervention after the event of injury." "560 (28.54 % ) individuals reported at least one injury during climbing resulting in a total number of 699 injuries. The most common body parts injured were feet (29.2% ), fi ngers (28.6% ) and legs (16.2% )." "Ligament injuries (36.8 % ), contusions (15.2 % ), fractures (15.0 % ) and tendon injuries (10.6 % ) were the most common injury types | Ligament and tendon injuries in the fingers, arms, hands, feet and legs. | • Difficulty level • Climbing experience • Climbing time per week during summer months • Climbing time per week during winter months • Sex • Age • BMI | • Difficulty level (p<0.01) • Climbing experience (p<0.01) • Climbing time per week during summer months (p<0.01) • Climbing time per week during winter months (p<0.01) • Sex, age, and BMI, were not associated with increased risk of injury. |
| Orth et al., (2022)  Cross-sectional study | Adult climbers, n=32 (15 injured, 17 control) / injured: 73% male, control: 81% male / injured: 30.99±1.6years, control: 30.89±2.4years / injured: 67.59±1.7kg, control 70.2±2.1 kg, NA, NA injured: 8 lead & bouldering, 7 bouldering, control: 8 lead & bouldering, 7 bouldering, 2 lead climbing / NA / NA / NA | Time elapsed since injury event limited to 6-9 months, diagnosis and treatment/advice for exercises must be given by a physical therapist | Previous climbing related injury to a single hand, diagnosed by a physiotherapist. | • Unilateral isometric finger flexor strength (maximal voulntary contraction (MVC) + rate of force development (RFD)) | When accounting for handedness, those with prior injury showed 7% reduced MVC (p=0.004) and 13% reduced RFD in injured hand (p=0.008). Nondominant hand: 11% weaker in MVC (p<0.001) and 12% weaker in RFD (p=0.02).. For uninjured group, no siginificant differences were found. |
| Paige et al., (1998) Cross-sectional retrospective study | Adult climbers, n= 398/ 80% male, 20% female/ mean age= 29 (range= 11-63)/ NA, NA, NA Traditional and sport climbers/ IRCRA grade range 1-29 / NA/ mean experience climbing= 7 years (range= 0.5-40 years) | Injury definition unclear, reported via  retrospective questionnaire. Both overuse and acute injuries reported. Incidence proportion of 63 injuries/100 participants in last 5 years. | Injuries referenced by anatomical location,  upper extremity injuries most common in  sports and traditional climbing. Finger  injuries represent 21% of injuries in trad  climbing and 33% in sports climbing. Difficult to discern category of injuries precisely e.g., overuse/overstrain versus. traumatic/fall-related injuries. | • Type of climbing • Climbing activity during injury | • "The ratio, however, of upper extremity to lower extremity injuries is greater in sport climbing, with a ratio of 4:1 (n = 49:12) for sport climbing and 2: 1 (n = 33: 16) for traditional climbing.  Among upper extremity injuries, the fingers are the most commonly injured structures. This is  especially true in sport climbing, where the ratio of finger injuries to all other injuries far exceeds  that seen in traditional climbing." • Lead climbing reported as a high risk factor: 79% of injuries occured whilst leading in sport  climbing, and 67% whilst leading during trad climbing. Relatively safer activities were belaying  and top roping. |
| Pieber et al. (2012) Cross-sectional study | Adult climbers, n= 193/ 69% male, 31% female/ males= 31.2±8.6 years, females= 29.4±5.6 years/ males= 72.7±8.2kg, 180.6±6.2cm, 22.2±1.8kg/m², female=: 57.3±7.2kg, 167.8±6.1cm, 20.3±2kg/m² Sport climbers and boulderers/ climbing intensity score: males= 2608.9±2788.8 ,females= 1695.6±1737.3/ NA/ males= 10.6±8.3 years, females= 6.6±5.5 | Acute injuries and overuse  injuries, identified by anatomical location, cause and diagnosis. Minor abrasions excluded.  67.4% of climbers reported a previous injury. 374 total injuries reported. Career incidence proportion of 194 injuries/100 participants | "The single most common differential diagnoses, jointly accounting for 56.7 % of all injuries, were strains and ruptures of annular ligaments or tendons of the fingers, epicondylitis of the elbow and sprains or fractures of the ankle joint." - Chronic finger arthropathies (7.5%) - Shoulder injuries (6.4%) - Spinal discomfort (5.3%) | • Age • Sex • Climbing intensity score | • Age group (29.5years vs. 23 years) injury incidence higher in older group (p=0.021) • Age group (39.7years vs. 29.5years)  no significant difference in injury • Sex: injury higher in males (p=0.032) • Climbing intensity score: injury  occured more often in higher intensity group (p=0.000) |
| Rohrbough et al., (2000) Cross-sectional study | Youth and adult elite competitive climbers in the USA (majority adult), n=42/ 83% male, 17% female/ mean age=25years (range: 13-40)/ NA, NA, NA Elite competitive climbing/ IRCRA grade 14 to 26/ NA/ mean years experience at elite level =4.5years | Injuries of upper extremities reported by location on visual diagram. Each participant also received a physical exam: "Pathology specifically tested for included bowstringing at the PIP joint and proximal phalanx, PIP collateral ligament laxity and tenderness, competence of the flexor digitorum superficialis (FDS) and flexor digitorum profundus (FDP) tendons, loss of active PIP extension, flexor tendon nodules, and triggering. If specific history implied symptoms of carpal tunnel syndrome, epicondylitis, or chronic shoulder pain, examination of these areas was done." Career incidence proportion= 300 injuries/100 participants | Collateral ligament injury (n=17, 40.5%), shoulder pain (n=14, 33.3%), bowstringing (n=11, 26.2%), flexor unit strain (n=11, 26.2%), A2 pulley pain (n=10, 23.8%), tendon nodule (n=10, 23.8%), medial epicondylitis (n=9, 21.4%), lateral epicondylitis (n=4, 9.5%), musculotendon junction pain (n=3, 7.1%), wrist undercling injury (n=3, 7.1%), and carpal tunnel syndrome (n=3, 7.1%). "The total number of injuries recorded were 126. Of these, 79 (63%) were in the hand, and 46 (37%) were elsewhere in the upper extremity." | • Age  • Sex • Total years climbing  • Top difficulty level climbed  • Years climbing at an elite level | • Age: significant for A2 pulley pain only (p=0.004) • Years of climbing experience: significant for history of medial epicondylitis only (p<0.0005) • Difficulty level climbing: no statistical association • Years climbing at an elite level: no statistical association • Sex: no statistical association |
| Runer et al. (2017) Prospective (one winter season) | Adult ice climbers, n= 70/ males= 68, females= 2/ 31.6±8.9years/ 74.8±8.9kg, 179.8±7.7cm, NA Ice climbing/ "beginner"-"expert" (WI1-WI7; 70% "intermediate")/ 4275 hours of exposure over winter season / 7.3±7.3years | Any physical complaint as a result of  training or competition, over the course of the winter season. Mostly acute and traumatic injuries reported. 9.8 injuries per 1000 exposure hours (42 injuries total during study period). | 14.3% of injuries reported were potentially  overuse in nature (unclear from study). These included joint sprains, ligament strains, and muscle strains. 73.8% were most likely traumatic  injuries (e.g., abrasion/contusion). | • Weather conditions • Safety • Overestimation of capacity • Physical preparedness  • Climbing level • Technical climbing error • Type of climb | • "Intermediate" athletes more likely to be injured (odds ratio, 2.55; 95% CI, 1.17-5.54;  P = 0.018) compared to "advanced"  climbers • 14.3% of injuries attributed to  "technical climbing error" • 7.1% injuries attributed to "overestimation of one's capacity" • 54.8% of injuries occured during lead climb |
| Schäfer et al., (1998) Cross-sectional study | Adult and youth climbers, n=112 / 29 female, 83 male / age 14-43years / NA, NA, NA  Indoor climbing / IRCRA grade ≥17 / training average 3 hours for 3,5d/wk / average 10 years of climbing | Any injury, or symptoms reported to the questionaire | Overuse: 19% finger, 7% spine, lower extremities 20%, upper extremities: 54%; most common overuse: epicondylopathies 33%, chronic finger overuse 28%, nerve compression syndrome 27%, muscular syndromes 23%, arthropathy shoulder 19%, arthropathy knee 14%, spine syndromes 13% | • Higher climbing ability  • High training intensity  • Bad planning of training  • Pull-ups in extended position  • Weak spine | • Prevention: training of antagonists (epicondylopathies) • Pull-ups should not be done from fully extended position  • Youth climbers should be trained carfully due to a weaker spine • Sufficient rest time between climbs reccomended (30min) No statistics reported |
| Schöffl et al., (2007) Longitudinal study (5years) | Adult and youth climbers, n=20, national team n=10, recreational n=10/ national team: 6 male 4 female, recreational: 8 male 2 female / national team: 16±1.6years (21±1.6years), recreational: 14±1.8years (19.9±1.9years) / NA, NA, BMI national team: pre 20.3±1.2kg/m²; post 21.8±1.5kg/m² recreational: pre 18.5±2.3kg/m²; post 21.6±3.1kg/m² Rock climbing / national team IRCRA grade pre 21 (range: 17-24); post 24 (range 21-28) recreational pre 12 (range 9-17); post 12 (range 8-18) / national team: pre 3.2±0.9; post 3.6±0.7 training units per week or pre 8.9±0.9; post 11.4±3.0hours per week; recreational: pre 1.3±0.7; post 1.5±1.4 training units/week or pre 3.5±2.4; post 2.3±2.0hours per week / national team: pre 5.9±3.5years; post 11.0±3.1years; recreational: pre 1.7±1.3years; post 5.6±2.4years | Osteoarthrosis was defined if a Kellgren-Lawrence grade ≥ 2 was present without definition of location; tendinitis. Radiographic stress reactions (subchrondral sclerosis/increased thickness of the epiphysis) stayed constant at 80% in national team (GJNT) while it increased from 20 to 30% in the recreational climbers (RC); detailled radiographic findings in table 4 within the study. | Pressure tenderness of finger base phalanx and of the medial epicondyle, osteoarthritis in fingers, stress reactions of the fingers. | • Years of climbing  • Participation in climbing competition • Hours of training/wk • Training units/wk • Climbing level | • Years of climbing (P < .01)  • Participation in climbing competitions (P < .01) • Hours of training per week (P < .01)  • Number of training units per week (P < .05) • Climbing level (P < .01) significant factors for development of radiographic stress reaction in all athletes (GJNT and RC) |
| Shahram et al., (2007) Cross-sectional retrospective survey | Male climbers from Iran, n=50/ 100% male/ NA, NA, NA, NA Boulderers, lead and top rope climbers/ NA/ NA/ NA | Any climbing related injury determined by clinical examination. 70% of climbers experienced injury. | "About half of the injuries were in the fingers (52.08%) and the rest were divided equally between the elbow-forearm (26.03%) and shoulder girdle (21.89%). The injury prevalence rate on the basis of the appearance in the fingers consists of the ring (43.07%), long (41.53%), index (12.3%) and small finger (3.07%) respectively." DIP joint dislocation (1.28%), PIP joint dislocation (1.28%), collateral ligament strain of PIP (16.66%), FDP muscle strain (16.66%), FDS muscle strain (19.23%), A2 annular pulley strain (8.97%), A2 annular pulley tear (1.28%), carpal tunnel syndrome (1.28%), lateral epicondylitis (6.41%), medial epicondylitis (2.56%), biceps brachi strain (7.69%), rotator cuff strain (14.1%), and rhomboid muscle strain (2.56%). | • Maximum climbing grade • Skillful hand • Type of climbing | • Maximum climbing grade (p=0.000) • Type of climbing (no significant association) |
| Stelzle et al., (2000)  Cross-sectional study | Adult and youth climbers, n=314 / 82 female, 232 male / age 14-60 / NA, NA, NA  Indoor climbing / IRCRA grade mean 13, male 7-29, female 4-25 / avergae 6 days/mo / average 2,5years | Injury=acute events caused by climbing and prevented sports for at least one day; overuse = chronic symptoms (pain and functional deficits) that started at least 6 months after climbing and lasted at least 6 weeks. 30% of climbers reported an overuse injury. | 61% finger, 11% elbow, 9% wrist, 5% shoulder, 5% spine, 5% knee, 2% ankle, 2% other | • Difficulty of climbing  • Preventive measures: using less injury-prone techniques (e.g. avoiding internal rotation knee) | Higher difficulty: more tendon, muscle and spine overuse but less joint overuse. No numbers reported. |
| van Middelkoop et al., (2015) Prospective Cohort (one year in length) | Climbers older than 12 years, n= 434/ 72.5%  male, 27.5% female/ 32.3±10.8 years/ NA, NA, 21.8±2.5kg/m² Sports climbers and boulderers/ 62% IRCRA grade of 16 or higher/ mean training exposure 16 hours per month/ mean 6.7±6.4 years experience | Upper extremity injuries identified over one year study period, defined as any damage on the body by climbing causing pain and/or disability. Incidence rate during one year 13.04 (95 %CI 11.86–14.30) injuries per 1 000 climbing hours. | Both acute and overuse injuries reported in  the upper extremities. Injuries reported by  anatomical location only: fingers (36%),  elbow (25.8%), wrist (19.5%), shoulder  (18.7%). | • Age • Sex • BMI • Education level • Work • Other sports activity • Climbing experience • Climbing time per month • Level of clmbing • Climbing type • Warm-up • Cool-down • Campus board climbing • Function DASH disability questionnaire • Finger strength (index, middle, ring) • Previous injury (<12 months) | Multivariate regression analysis • Higher age (OR 1.03, 95 % CIs 1.01; 1.05)  • Performing a cool-down (OR 2.02, 95 % CIs 1.28; 3.18)  • Training with campus board (OR 2.48, 95 % CIs 1.23; 5.02) • Middle finger strength (OR 1.12, 95 % CIs 1.05;1.18)  • Previous injuries (OR 3.12, 95 % CIs 2.01; 4.83)  Univariate analysis • Climbing level (6a+) (OR 2.03, 95% CIs 1.34; 3.07) • Bouldering (OR 2.02, 95CIs 1.01; 4.05) |
| Wright et al., (2001) Cross-sectional study | Youth and adult indoor climbers, n=295/ range <20 to 35+years/ NA/ NA, NA, NA Sport climbers (bouldering and lead)/ NA/ NA/ Range <10 to 10+ years | Overuse injuries sustained whilst climbing indoors, defined as "strain" injuries i.e., "non-traumatic".  131 climbers (44%) sustained an overuse injury. 57 climbers suffered injuries at more than one site. Fingers were injured most prevalently (32% of respondents). Six climbers injured their knees, and four injured their back. | Not specified and only referred to by anatomical location (fingers, upper limb, knees, and back). | • Sex • Preferred activity (boulder, lead, top rope) • Climbing grade • Age group • Years climbing • Visits per annum | • Male sex (p=0.009) • Bouldering vs. top roping (p=0.001) • Bouldering/leading (together) vs. top roping (p<0.0005) • Lead grade (p<0.0005) • Bouldering (p<0.0005) • Age group (p=0.576) • Years experience (p=0.006) • Visits per annum (p=0.016) |
| Zielinski et al., (2021) Cross-sectional study, retrospective survey | Climbers on climbing walls in Lublin, Poland, n=113/ 88 males and 35 females/ 30±8 years/ NA, NA, NA Probably sport climbers (lead climbers and boulderers)/ IRCRA grade 16 to 24/ NA/ NA | Low back pain: "Oswestry Low Back Pain Disability Index was used to assess the severity and frequency of pain, and the degree of reduced function caused by lumbar spine pain. The questionnaire examines the effects of back pain in 10 planes: pain intensity, self-service, carrying, walking, sitting, standing, sleeping, socializing, travelling, and changes in pain intensity." Mild back pain prevalence was 26%. | Low back pain | • Climbing type (lead vs. Boulder) • Climbing grade | • Bouldering associated with higher risk of mild low back pain (30% vs. 10%) (p<0.05) • Higher prevalence of low back pain in elite climbers (intermediate=22%, advanced=19%, elite=50%), although no statistical significance reported. |
| IRCRA, International Rock Climbing Reserach Association; NA, not available; BMI, Body Mass Index; OR, odds ratio; CI, Confidence Interval; DIP, distal interphalangeal joint; PIP, proximal interphalangeal joint; FDS, flexor digitorum superficialis; FDP, flexor digitorum profundus;  MVC, maxium voluntary contraction; RFD, rate of force development; BME, bone marrow edemas; UIAA, Union Internationale des Associations d'Alpinisme; IP, incidence proportion; GJNT, German Junior National Team; RC, recreational climbers; CRIH, climbing related injuries of the hand | | | | | |
